# Dynamical Transitions in a Pollination–Herbivory Interaction: A Conflict between Mutualism and Antagonism

**DOI:** 10.1371/journal.pone.0117964

**Published:** 2015-02-20

**Authors:** Tomás A. Revilla, Francisco Encinas–Viso

**Affiliations:** 1 Centre for Biodiversity Theory and Modelling, Station d’Ecologie Expérimentale du Centre National de la Recherche Scientifique à Moulis, Moulis, France; 2 Community and Conservation Ecology Group, Centre for Ecological and Evolutionary Studies, University of Groningen, Groningen, The Netherlands; Universitat Pompeu Fabra, SPAIN

## Abstract

Plant-pollinator associations are often seen as purely mutualistic, while in reality they can be more complex. Indeed they may also display a diverse array of antagonistic interactions, such as competition and victim–exploiter interactions. In some cases mutualistic and antagonistic interactions are carried-out by the same species but at different life-stages. As a consequence, population structure affects the balance of inter-specific associations, a topic that is receiving increased attention. In this paper, we developed a model that captures the basic features of the interaction between a flowering plant and an insect with a larval stage that feeds on the plant’s vegetative tissues (e.g. leaves) and an adult pollinator stage. Our model is able to display a rich set of dynamics, the most remarkable of which involves victim–exploiter oscillations that allow plants to attain abundances above their carrying capacities and the periodic alternation between states dominated by mutualism or antagonism. Our study indicates that changes in the insect’s life cycle can modify the balance between mutualism and antagonism, causing important qualitative changes in the interaction dynamics. These changes in the life cycle could be caused by a variety of external drivers, such as temperature, plant nutrients, pesticides and changes in the diet of adult pollinators.

## Introduction

*Il faut bien que je supporte deux ou trois chenilles si je veux connaître les papillons*

Le Petit Prince, Chapitre IX – Antoine de Saint-Exupéry

Mutualism can be broadly defined as cooperation between different species [1]. In mutualistic interactions typically there are benefits and costs, in terms of resources, energy and time devoted to them, but the net outcome is (+,+) in the final balance. However, there can be other kinds of costs, concerning detrimental interactions that run in parallel with mutualism, such as predation, parasitism or competition, involving the same parties. Moreover, some of these antagonistic interactions (e.g. competition) seem to be important for the evolution and stability of mutualism [2]. In general, these costs have important consequences at the population and community level because the net outcome of an interspecific association can turn out beneficial or detrimental and more interestingly, variable [3]. Variable interactions challenge the view that ecological communities are structured by well defined interactions at the species level such as competition (−,−), victim-exploiter (−,+) or mutualism (+,+).

Pollination is one of the most important mutualisms occurring between plants and animals. This form of trading resources for services greatly explains the evolutionary success of flowering plants in almost all terrestrial systems. It is responsible for the well being of ecosystem services. During the larval stage of many insect pollinators, such as Lepidopterans (butterflies and moths), the larvae feed on plant leaves to mature and become adult pollinators [4–7]. These ontogenetic diet shifts [8] are very common and important in understanding the ecological and evolutionary dynamics of plant–animal mutualisms. Interestingly, in some cases larvae feed on the same plant species that they will pollinate as adults [6, 9]. This shows that in several cases mutualistic and antagonistic interactions are exerted by the same species, and a potential conflict arises for the plant, between the benefits of mutualism and the costs of herbivory. One of the best known examples is the interaction between tobacco plants (*Nicotiana* *attenuata*) and the hawkmoth (*Manduca* *sexta*) [10, 11], whose larva is commonly called the tobacco hornworm. There are other examples of this type of interaction in the genus *Manduca* (Sphingidae), such as between the tomato plant (*Lycopersicon esculentum*) and the five-spotted hawkmoth (*Manduca quinquemaculata*) [12]. These larvae have received a lot of attention due to their negative effects on agricultural crops [13].

The interaction between *Manduca sexta* and *Datura wrightii* (Solanacea) [6, 14] is another good example illustrating the costs and benefits of pollination mutualisms [6]. *D. wrightii* provides high volumes of nectar and seems to depend heavily on the pollination service by *M. sexta* adults [14]. However, *M. sexta* larvae, which feed on *D. wrightii* vegetative tissue, can have severe negative effects on plant fitness [15, 16]. We could assume that the benefits of pollination might outweigh the costs of herbivory for this mutualism to be relatively viable. The question is what are the conditions, in terms of benefits (pollination) and costs (herbivory), for this mutualistic interaction to be stable?

In the pollination–herbivory cases mentioned previously the benefits and costs for the plant are clearly differentiated. This is because the role of an insect as a pollinator or herbivore depends on the stage in its life cycle [17]. Thus, whether mutualism or herbivory dominates the interaction is dependent on insect abundance and its population structure. In other words the *cost:benefit* ratio must be positively related with the insect’s *larva:adult* ratio. For a hypothetical scenario in which the costs of herbivory (−) and the benefits of pollination (+) are balanced for the plant (0), an increase in larval abundance relative to adults should bias the relationship towards a victim-exploiter one (−,+). Whereas an increase in adult abundance relative to larvae should bias the relationship towards mutualism (+,+). Under equilibrium conditions, one would expect transitions (bifurcations) from (−,+) to (0,+) to (+,+) and vice-versa as relevant parameters affecting the plant and the insect life-histories vary, such as flower production, mortalities or larvae maturation rates. However, under dynamic scenarios the outcome may be more complex: a victim–exploiter state (−,+) enhances larva development into pollinating adults, but this tips the interaction into a mutualism (+,+), which in turn contributes greater production of larva leading back to a victim–exploiter state (−,+). This raises the possibility of feedback between the plant–insect interaction and insect population structure, which can potentially lead to periodic alternation between mutualism and herbivory. Thus, when non-equilibrium dynamics are involved, questions concerning the overall nature (positive, neutral or negative) of mixed interactions may not have simple answers.

In this article we study the feedback between insect population structure, pollination and herbivory. We want to understand how the balance between costs (herbivory) and benefits (pollination) affects the interaction between plants (e.g. *D. wrightii*) and herbivore–pollinator insects (e.g. *M. sexta*)? Also what role does insect development have in this balance and on the resulting dynamics? We use a mathematical model which considers two different resources provided by the same plant species, nectar and vegetative tissues. Nectar consumption benefits the plant in the form of fertilized ovules, and consumption of vegetative tissues by larvae causes a cost. Our model predicts that the balance between mutualism and antagonism, and the long term stability of the plant–insect association, can be greatly affected by changes in larval development rates, as well as by changes in the diet of adult pollinators.

## Methods

**Fig 1:**
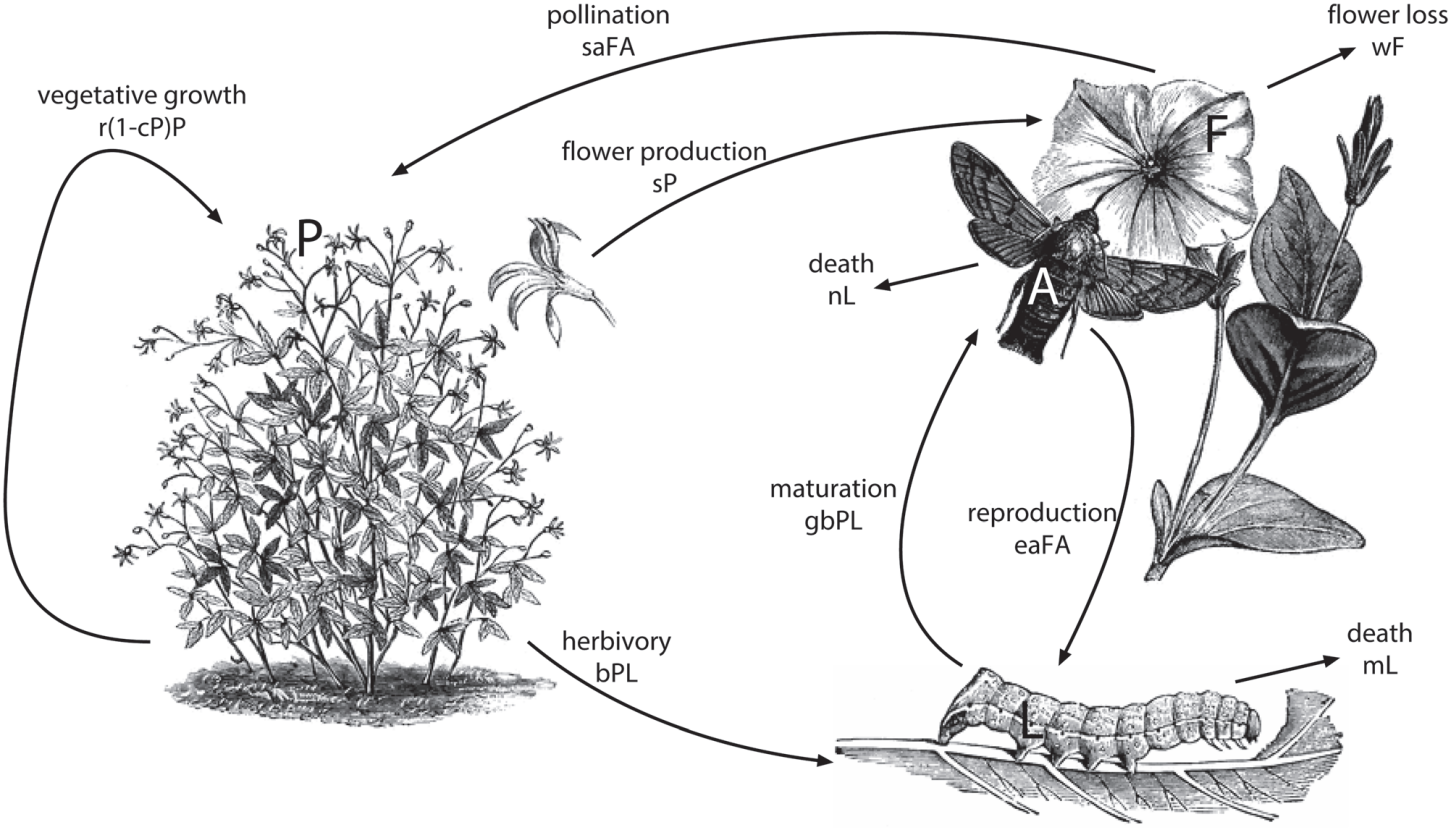
Interaction mechanism between plants (*P*), flowers (*F*), larva (*L*), adult insects (*A*) and associated biomass flows. Clipart sources: http://etc.usf.edu/clipart/

Our model concerns the dynamics of the interaction between a plant and an insect. The insect life cycle comprises an adult phase that pollinates the flowers and a larval phase that feed on non-reproductive tissues of the same plant. Adults oviposit on the same species that they pollinate (e.g. *D. wrightii – M. sexta* interaction). Let denote the biomass densities of the plant, the larva, and the adult insect with *P*, *L* and *A* respectively. An additional variable, the total biomass of flowers *F*, enables the mutualism by providing resources to the insect (nectar), and by collecting services for the plant (pollination). The relationship is *facultative–obligatory*. In the absence of pollination, plant biomass persists by vegetative growth (e.g. root, stem and leaf biomass are being constantly renewed). For the sake of simplicity and because we want to focus on the plant–insect interaction, we describe vegetative growth using a logistic growth rate, a choice that is empirically justified for tobacco plants [18]. In the absence of the plant, however, the insect always goes extinct because larval development relies exclusively on herbivory, even if adults pollinate other plant species. This is based on the biology of *M. sexta* [6]. The mechanism of interaction between these four variables (*P*, *L*, *A*, *F*), as shown in Fig. 1, is described by the following system of ordinary differential equations (ODE):

$$\begin{array}{c} \frac{dP}{dt}\;=\;rP\left(1-cP\right)+\sigma aFA-bPL\\ \frac{dF}{dt}\;=\;sP-wF-aFA\\ \frac{dL}{dt}\;=\;\epsilon aFA+gA-\gamma bPL-mL\\ \frac{dA}{dt}\;=\;\gamma bPL-nA \end{array}$$ (1)

where *r*: plant intrinsic growth rate, *c*: plant intra-specific self-regulation coefficient (also the inverse its carrying capacity), *a*: pollination rate, *b*: herbivory rate, *s*: flower production rate, *w*: flower decay rate, *m*, *n*: larva and adult mortality rates, *σ*: plant pollination efficiency ratio, *ε*: adult consumption efficiency ratio. Like *ε*, parameter *γ* is also a consumption efficiency ratio, but we will call it the maturation rate for brevity since we will refer to it frequently. Our model assumes that pollination leads to flower closure [19], causing resource limitation for adult insects. Parameter *g* represents a reproduction rate resulting from the pollination of other plants species, which we do not model explicitly. Most of our results are for *g* = 0.

We now consider the fact that flowers are ephemeral compared with the life cycles of plants and insects. In other words, some variables (*P*, *L*, *A*) have slower dynamics, and others (*F*) are fast [20]. Given the near constancy of plants and animals in the flower equation of (1), we can predict that flowers will approach a quasi-steady-state (or quasi-equilibrium) biomass *F* ≈ *sP*/(*w* + *aA*), before *P*, *L* and *A* can vary appreciably. Substituting the quasi-steady-state biomass in system (1) we arrive at:

$$\begin{array}{c} \frac{dP}{dt}\;=\;rP\left(1-cP\right)+\sigma \left[\frac{asA}{w+aA}\right]P-bPL\\ \frac{dL}{dt}\;=\;\epsilon \left[\frac{asP}{w+aA}\right]A+gA-\gamma bPL-mL\\ \frac{dA}{dt}\;=\;\gamma bPL-nA \end{array}$$ (2)

In system (2) the quantities in square brackets can be regarded as functional responses. Plant benefits saturate with adult pollinator biomass, i.e. pollination exhibits diminishing returns. The functional response for the insects is linear in the plant biomass, but is affected by intraspecific competition [21] for mutualistic resources.

We non-dimensionalized this model to reduce the parameter space from 12 to 9 parameters, by casting biomasses with respect to the plant’s carrying capacity (1/*c*) and time in units of plant biomass renewal time (1/*r*). This results in a PLA (plant, larva, adult) scaled model:

$$\begin{array}{c} \begin{array}{c} \frac{dx}{d\tau }\;=\;x\left(1-x\right)+\sigma \frac{\alpha z}{\eta +z}x-\beta xy\\ \frac{dy}{d\tau }\;=\;\epsilon \frac{\alpha x}{\eta +z}z+\phi z-\gamma \beta xy-\mu y\\ \frac{dz}{d\tau }\;=\;\gamma \beta xy-\nu z \end{array} \end{array}$$ (3)

Table 1 lists the relevant transformations.

**Table 1:** Variables and parameters.

Symbol	Description	Value	*c* = 0.01, *r* = 0.05
*x* = *cP*, *y* = *cL*, *z* = *cA*	plant, larval and adult biomass	variable	
*τ* = *rt*	time	variable	
*α* = *s*/*r*	asymptotic pollination rate	5	*s* = 0.25
*η* = *wc*/*a*	half-saturation constant of pollination	0.1	*w* = 0.5 & *a* = 0.05
*β* = *b*/*rc*	herbivory rate	0 to 100	*b* = 0 to 0.05
*μ* = *m*/*r*	larva mortality rate	1	*m* = 0.05
*ν* = *n*/*r*	adult mortality rate	2	*n* = 0.1
*ϕ* = *g*/*r*	insect intrinsic reproduction rate	0 or 1	*g* = 0 or 0.05
*σ*	plant pollination conversion ratio	5	
*ε*	insect pollination conversion ratio	0.5	
*γ*	maturation rate (herbivory conversion ratio)	0 to 0.1	

Variables and parameters of the scaled PLA model (3) and values used for numerical analyses. The last column shows a corresponding set of parameter values in the unscaled version of the same model (2), for plant carrying capacities of *c* ^−1^ = 100 biomass units, and *r* ^−1^ = 20 time units.

There is an important clarification to make concerning the nature and scales of the conversion efficiency ratios *σ*, *ε* involved in pollination, and *γ* for herbivory and maturation. This has to do with the fact that flowers *per se* are not resources or services, but *organs* that enable the mutualism to take place, and they mean different things in terms of biomass production for plants and animals. For insects, the yield of pollination is thermodynamically constrained. First of all, a given biomass *F* of flowers contains an amount of nectar that is necessarily less than *F*. More importantly, part of this nectar is devoted to survival, or wasted, leaving even less for reproduction. Similarly, not all the biomass consumed by larvae will contribute to their maturation to adult. *Ergo* *ε* < 1, *γ* < 1. Regarding the returns from pollination for the plants, the situation is very different. Each flower harbors a large number of ovules, thus a potentially large number of seeds [22], each of which will increase in biomass by consuming resources not considered by our model (e.g. nutrients, light). Consequently, a given biomass of pollinated flowers can produce a larger biomass of mature plants, making *σ* larger than 1.

The PLA model (3) has many parameters. However, here we focus on herbivory rates (*β*) and larvae maturation (*γ*) because increasing *β* turns the net balance interaction towards antagonism, whereas increasing *γ* shifts insect population structure towards the adult phase, turning the net balance towards mutualism. Both parameters also relate to the state variables at equilibrium (i.e. *z*/*y* = *βγx*/*ν* in (3) for *dz*/*dτ* = 0). We studied the joint effects of varying *β* and *γ* numerically (parameter values in Table 1) using XPPAUT [23]. ODE were integrated using Matlab [24] or GNU/Octave [25]. We also present a simplified graphical analysis of our model, in order to explain how different dynamics can arise, by varying other parameters. The source codes supporting these results are provided as supplementary material (S1 File).

## Results

### Numerical results

**Fig 2:**
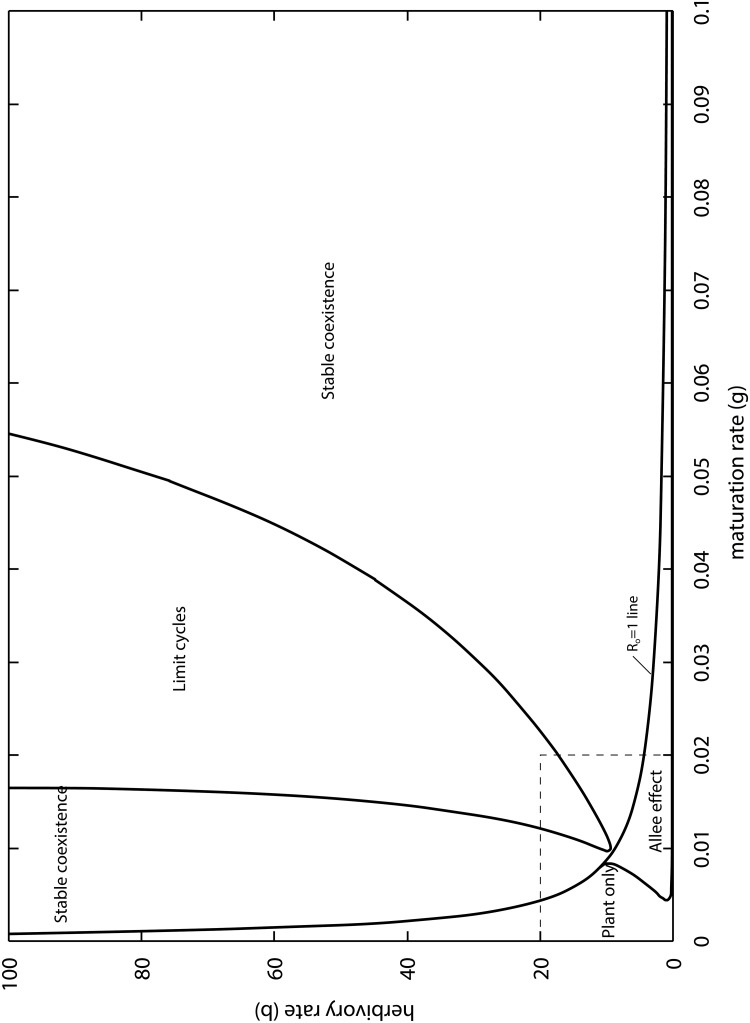
Outcomes of the PLA model as a function of the larval maturation and herbivory rates for specialist pollinators (*ϕ* = 0). The rectangular region in the bottom left is analyzed with more detail in S1 File.

Fig. 2 shows interaction outcomes of the PLA model, as a function of *β* and *γ* for specialist pollinators (*ϕ* = 0). This parameter space is divided by a decreasing *R*_*o*_ = 1 line that indicates whether or not insects can invade when rare. *R*_*o*_ is defined as (see derivation in S1 File):

$$\begin{array}{c} R_{o}=\frac{\epsilon \alpha \gamma \beta }{\eta \nu (\mu +\gamma \beta )} \end{array}$$ (4)

and we call it the *basic reproductive number*, according to the argument that follows. Consider the following in system (3): if the plant is at carrying capacity (*x* = 1), and is invaded by a very small number of adult insects (*z* ≈ 0), the average number of larvae produced by a single adult in a given instant is *εαx*/(*η*+*z*) ≈ *εα*/*η*, and during its life-time (*ν* ^−1^) it is *εα*/*ην*. Larvae die at the rate *μ*, or mature with a rate equal to *γβx* = *γβ*, per larva. Thus, the probability of larvae becoming adults rather than dying is *γβ*/(*μ*+*γβ*). Multiplying the life-time contribution of an adult by this probability gives the expected number of new adults replacing one adult per generation during an invasion (*R*_*o*_). More formally, *R*_*o*_ is the expected number of adult-insect-grams replacing one adult-insect-gram per generation (assuming a constant mass-per-individual ratio).

Below the *R*_*o*_ = 1 line, small insect populations cannot replace themselves (*R*_*o*_ < 1) and two outcomes are possible. If the maturation rate is too low, the plant only equilibrium (*x* = 1, *y* = *z* = 0) is globally stable and plant–insect coexistence is impossible for all initial conditions. If the maturation rate is large enough, stable coexistence is possible, but only if the initial plant and insect biomass are large enough. This is expected in models where at least one species, here the insect, is an obligate mutualist. In this region of the space of parameters, the growth of small insect populations increases with population size, a phenomenon called the Allee effect [26].

Above the *R*_*o*_ = 1 line the plant only equilibrium is always unstable against the invasion of small insect populations (*R*_*o*_ > 1). Plants and insects can coexist in a stable equilibrium or via limit cycles (stable oscillations). The zone of limit cycles occurs for intermediate values of the maturation rate (*γ*) and it widens with rate of herbivory (*β*).

Plant equilibrium when coexisting with insects can be above or below the carrying capacity (*x* = 1). When above carrying capacity the net result of the interaction is a mutualism (+,+). While in the second case we have antagonism, more specifically net herbivory (−,+). As it would be expected, increasing herbivory rates (*β*) shifts this net balance towards antagonism (low plant biomass), while decreasing it shifts the balance towards mutualism (high plant biomass). The quantitative response to increases in the maturation rate (*γ*) is more complex however (see the bifurcation plot in S1 File).

**Fig 3:**
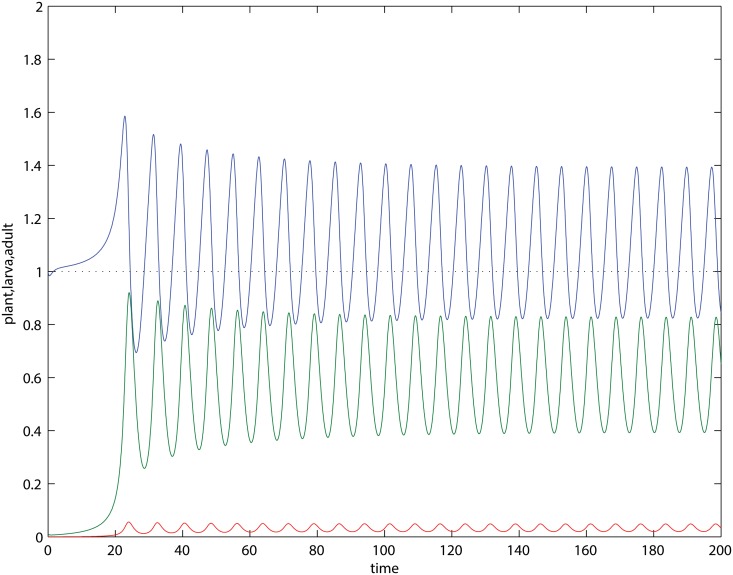
Limit cycles in the PLA model (3). Plant biomass alternates above and below the carrying capacity (dotted line). Parameters as in Table 1, with *γ* = 0.01, *β* = 10. Blue:plant, green:larva, red:adult.

Given that there is herbivory, we encounter victim–exploiter oscillations. However, the oscillations in the PLA model are special in the sense that the plant can attain maximum biomasses above the carrying capacity (*x* > 1). For an example see Fig. 3. Instead of a stable balance between antagonism and mutualism, we can say that the outcome in Fig. 3 is a periodic alternation of both cases. This is not seen in simple victim–exploiter models, where oscillations are always below the victim’s carrying capacity [27, 28]. The relative position of the cycles along the plant axis is also affected by herbivory: if *β* decreases (increases), plant maxima and minima will increase (decrease) in Fig. 3 (see bifurcation plot in S1 File). In some cases the entire plant cycle (maxima and minima) ends above the carrying capacity if *β* is low enough (see S1 File), but further decrease causes damped oscillations. We also found examples in which coexistence can be stable or lead to limit cycles depending on the initial conditions (see example in S1 File), but this happens in a very restrictive region in the space of parameters (see bifurcation plot in S1 File). Limit cycles can also cross the plant’s carrying capacity under the original interaction mechanism (1), which does not assume the steady–state in the flowers (see S1 File, using parameters in the last column of Table 1).

**Fig 4:**
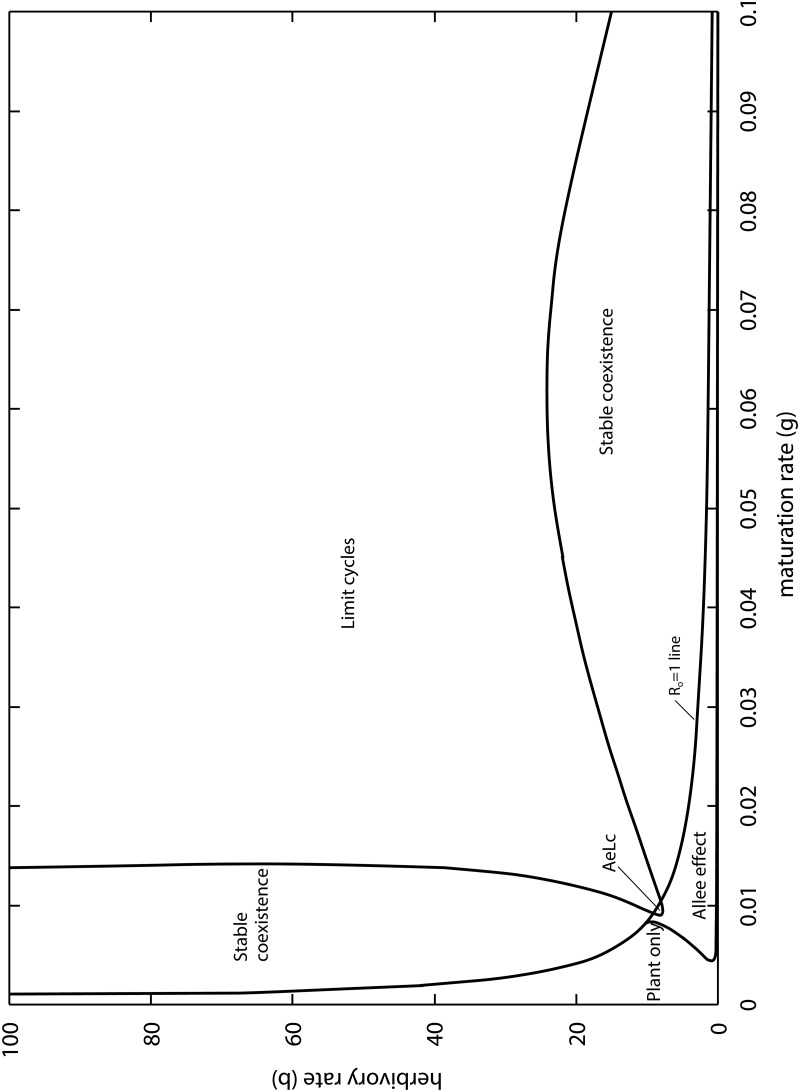
Outcomes of the PLA model as a function of the larval maturation and herbivory rates for generalist pollinators (*ϕ* = 1). AeLc: intersection of the Allee effect and Limit cycle zones.

Fig. 4 shows the *β* vs *γ* parameter space of the model when the adults are more generalist. The relative positions of the plant-only, Allee effect, and coexistence regions are similar to the case of specialist pollinators (Fig. 2). However, the region of limit cycles is much larger. The *R*_0_ = 1 line is closer to the origin, because the expression for *R*_0_ is now (see derivation in S1 File):

$$\begin{array}{c} R_{0}=\frac{(\epsilon \alpha +\phi \eta )\gamma \beta }{\eta \nu (\mu +\gamma \beta )} \end{array}$$ (5)

In other words, this means that the more generalist the adult pollinators (larger *ϕ*), the more likely they can invade when rare. There is also a small overlap between the Allee effect and limit cycle regions, i.e. parameter combinations for which the long term outcome could be insect extinction or plant–insect oscillations, depending on the initial conditions.

### Graphical analysis

The general features of the interaction can be studied by phase-plane analysis. To make this easier, we collapsed the three-dimensional PLA model into a two-dimensional plant–larva (PL) model, by assuming that adults are extremely short lived compared with plants and larvae (see resulting ODE in S1 File). The closest realization of this assumption could be *Manduca sexta*, which has a larval stage of approximately 20–25 days and adult stages of around 7 days [29, 30]. For a given parametrization (Table 1), the PL model has the same equilibria as the PLA model, but not the exact same global dynamics due to the alteration of time scales. Yet, this simplification provides insights about the outcomes displayed in Figs. 2 and 4.

**Fig 5:**
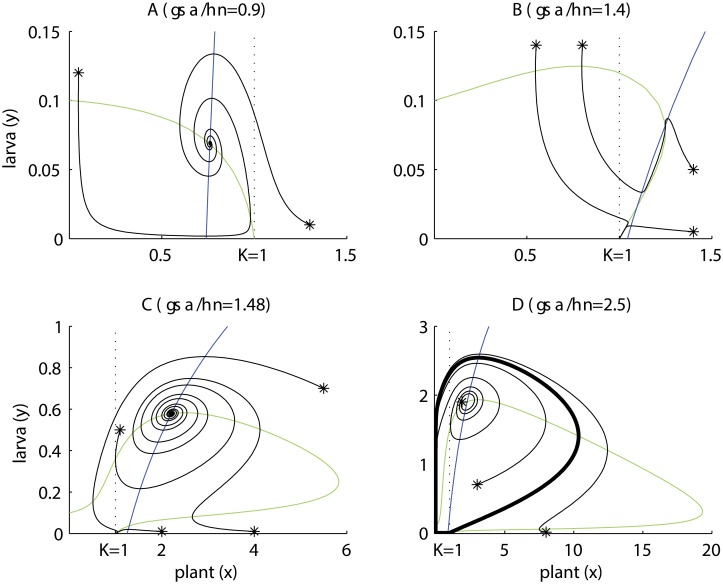
Dynamics of the simplified version of the PLA model. Plant isoclines in green and larva isoclines in blue. Several trajectories are shown (starting with *). The dotted line at *x* = 1 is the plant’s carrying capacity. When *γσα*/*ην* < 1 the plant’s isocline always decreases, when *γσα*/*ην* > 1, it bulges above the carrying capacity and displays a hump. (A) Damped oscillations leading to globally stable coexistence dominated by antagonism (victim–exploiter). (B) The isoclines intersect as a locally stable mutualistic equilibrium and as a saddle point. Insects can coexist with the plant or go extinct depending on the initial conditions. (C) This is similar to case (B), however, a stable mutualism occurs only after damped oscillations or the insect go extinct, depending on the initial conditions. (D) Here the system develops oscillations approaching a limit cycle (thick loop), which creates a periodic alternation between mutualism and antagonism. Common parameters in all panels are *β* = 10, *η* = 0.1, *μ* = 1, *ϕ* = 0. For the other parameters; in (A): *σ* = 3, *ε* = 0.7, *α* = 3, *γ* = 0.02, *ν* = 2; in (B): *σ* = 2.1, *ε* = 0.21, *α* = 2, *γ* = 0.05, *ν* = 1.5; in (C): *σ* = 3.7, *ε* = 0.2, *α* = 3, *γ* = 0.02, *ν* = 1.5; in (D): *σ* = 5, *ε* = 0.3, *α* = 5, *γ* = 0.02, *ν* = 2.

Fig. 5 shows representative examples of plant and larva isoclines (i.e. non-trivial nullclines) and coexistence equilibria (intersections). Isocline properties are analytically justified (see S1 File and supplemented [31] worksheet). The local dynamics around equilibria depends on the eigenvalues of the jacobian matrix of the PL model at the equilibrium. However, the highly non-linear nature of the PL model (see S1 File), makes it pointless to try infer the signs of the eigenvalues by analytical means (except for trivial and plant-only equilibrium). Thus, we propose to use to local geometry of isocline intersections to infer local stability [32]. Plant isoclines take two main forms:

$$\begin{array}{c} \left\{\begin{array}{cc} \gamma \sigma \alpha <\eta \nu & \text{the}\;\text{isocline}\;\text{lies}\;\text{entirely}\;\text{below}(\text{to}\;\text{the}\;\text{left}\;\text{of})\text{the}\;\text{carrying}\;\text{capacity}\\ \gamma \sigma \alpha >\eta \nu & \text{parts}\;\text{of}\;\text{the}\;\text{isocline}\;\text{lie}\;\text{above}(\text{to}\;\text{the}\;\text{right}\;\text{of})\text{the}\;\text{carrying}\;\text{capacity} \end{array}\right. \end{array}$$ (6)

In both cases, plants grow between the isocline and the axes, and decrease otherwise. Larva isoclines are simpler, they start in the plant axis and bend towards the right when insects tend towards specialization (*ϕ* < *ν*), as shown by Fig. 5. When insects tend towards generalism (*ϕ* > *ν*), their isoclines increase rapidly upwards like the letter “J” (not shown here, see S1 File). Insects grow below and right of the larva isocline, and decrease otherwise.

The *γσα* < *ην* case in Fig. 5A illustrates scenarios in which pollination rates (*α*), plant benefits (*σ*), adult pollinator lifetimes (1/*ν*) and larva-to-adult transition rates (*γ*) are low. The plant’s isocline is a decreasing curve crossing the plant’s axis at its carrying capacity K (*x* = 1, *y* = 0). The intersection with the larva isocline creates a globally stable equilibrium, approached by oscillations of decreasing amplitude. The local stability of this equilibrium can be explained partly by the geometry of the intersection: Fig. 5A shows that if plants increase (decrease) above (below) the intersection point, while keeping the insect density fixed, they enter a zone of negative (positive) growth; and the same behavior holds for the insects while keeping the plants fixed. In ecological terms, both species are self-limited around the equilibrium, a strong indication of stability [32]. Together with the fact that the trivial (*x* = 0, *y* = 0) and carrying capacity equilibrium (*x* = 1, *y* = 0) are saddle points, we conclude that plants and insects achieve a globally stable equilibrium after a period of transient oscillations (provided that insects are viable, e.g. *β*, *γ*, *ε* are large enough). This equilibrium is demographically unfavorable for the plant because its biomass lies below the carrying capacity (*x* < 1). Indeed, for extreme scenarios of negligible plant pollination benefits (i.e. *α* and/or *σ* tend to zero), the plant’s isocline approximates a straight line with a negative slope, like the isocline of a logistic prey in a Lotka–Volterra model, which is well known to cause damped oscillations [32].

The *γσα* > *ην* case in Figs. 5B,C,D cover scenarios in which pollination rates (*α*), pollination benefits (*σ*), adult pollinator lifetimes (1/*ν*) and larva-to-adult (harm-to-benefit) transition rates (*γ*) are high. One part of the plant’s isocline lies above the carrying capacity, which means that coexistence equilibria with plant biomass larger than the carrying capacity (*x* > 1) are possible, and this is favorable for the plant. Fig. 5B, shows and example where the larva isocline intersects the plant’s isocline twice above the carrying capacity. One intersection is a locally stable coexistence equilibrium, whereas the other intersection is a saddle point. The saddle point belongs to a boundary that separates regions of initial conditions leading to insect persistence or extinction. This can explain the Allee effect, i.e. insect growth rates increase (go from negative to positive) with insect density when insect populations are very small.

As the second inequality of (6) widens (*γσα* ≫ *ην*), the plant’s isocline takes a mushroom-like shape (or “anvil” or letter “Ω”), as in Fig. 5C,D. The plant’s isocline displays a very prominent “hump”, like in the prey isocline of the Rosenzweig–MacArthur model [27]. As a “rule of thumb”, intersections at the right of the hump would lead to damped oscillations, for the reasons explained before (Fig. 5A, for *γσα* < *ην*). Also as a “rule of thumb”, intersections at the left of the hump (like in Fig. 5C,D) are expected to result in reduced stability. This is because a small increase (decrease) along the plant’s axis leaves the plant at the growing (decreasing) side of its isocline, promoting further increase (decrease). This means that plants do not experience self-limitation, which is an indication of instability [32], and we infer that oscillations will not vanish. Fig. 5D shows an example where an intersection at the left of the hump causes instability, leading to limit cycles. However, Fig. 5C shows an exception of this prediction (the intersection is stable). In both examples the intersection occurs above the plants carrying capacity, thus revealing oscillations alternating above and below the plant’s carrying capacity. We want to stress one more time, that these predictions based on isocline intersection configurations (left vs right of the hump) must be taken as “rules of thumb”.

Fig. 5C also reveals an important consequence of the dual interaction between the plant and the insect. As we can see, the presence of a saddle point leads to the Allee effect explained before. But this figure also shows that large larval densities can lead to insect extinction. This can be explained by the fact that at large initial densities, the larva overexploits the plant, and this is followed by an insect population crash from which it cannot recover due to the Allee effect.

As *γ*, *σ*, *α* increase and/or *η*, *ν* decrease more and more, the decreasing segment of the plant isocline (the part at the right of the hump) approximates a decreasing line (actually a straight asymptotic line, see S1 File), while the rest of the isocline is pushed closer and closer to the axes. In other words, when pollination rates (*α*), benefits (*σ*), adult lifetimes (1/*ν*) and larva development rates (*γ*) increase, plant isoclines would resemble the isocline of a logistic prey, with a “pseudo” carrying capacity (the rightmost extent of the isocline) larger than the intrinsic carrying capacity (*x* = 1). Fig. 5D is an example of this. These conditions would promote stable coexistence with large plant equilibrium biomasses.

## Discussion

We developed a plant–insect model that considers two interaction types, pollination and herbivory. Ours belongs to a class of models [33, 34] in which balances between costs and benefits cause continuous variation in interaction strengths, as well as transitions among interaction types (mutualism, predation, competition). In our particular case, interaction types depend on the stage of the insect’s life cycle, as inspired by the interaction between *M. sexta* and *D. wrightii* [6, 14] or between *M. sexta* and *N. attenuata* [10]. There are many other examples of pollination–herbivory in Lepidopterans, where adult butterflies pollinate the same plants exploited by their larvae [5, 7]. We assign antagonistic and mutualistic roles to larva and adult insect stages respectively, which enable us to study the consequences of ontogenetic changes on the dynamics of plant–insect associations, a topic that is receiving increased attention [8, 17]. Our model could be generalized to other scenarios, in which drastic ontogenetic niche shifts cause the separation of benefits and costs in time and space. However, it excludes cases like the yucca/yucca moth interaction [35] where adult pollinated ovules face larval predation, i.e. benefits themselves are deducted.

Instead of using species biomasses as resource and service proxies [34], we consider a mechanism (1) that treats resources more explicitly [36]. We use flowers as a direct proxy of resource availability, by assuming a uniform volume of nectar per flower. Nectar consumption by insects is concomitant with service exploitation by the plants (pollination), based on the assumption that flowers contain uniform numbers of ovules. Pollination also leads to flower closure [19], making them limiting resources. Flowers are ephemeral compared with plants and insects, so we consider that they attain a steady-state between production and disappearance. As a result, the dynamics is stated only in terms of plant, larva and adult populations, i.e. the PLA model (3). The feasibility of the results described by our analysis depends on several parameters. The consumption, mortalities and growth rates, and the carrying capacities (e.g. *a*, *b*, *m*, *n* and *r*, *c* in the fourth column of Table 1), have values close to the ranges considered by other models [34, 37]. Oscillations, for example, require large herbivory rates, but this is usual for *M. sexta* [15].

### Mutualism–antagonism cycles

The PLA model displays plant–insect coexistence for any combination of (non-trivial) initial conditions where insects can invade when rare (*R*_*o*_ > 1). Coexistence is also possible where insects cannot invade when rare (*R*_*o*_ < 1), but this requires high initial biomasses of plants and insects (Allee effect). Coexistence can take the form of a stable equilibrium, but it can also take the form of stable oscillations, i.e. limit cycles.

Previous models combining mutualism and antagonism predict oscillations, but they are transient ones [35, 38], or the limit cycles occur entirely below the plant’s carrying capacity [39]. We have good reasons to conclude that the cycles are herbivory driven and not simply a consequence of the PLA model having many variables and non-linearities. First of all, limit cycles require herbivory rates (*β*) to be large enough. Second, given limit cycles, an increase in the maturation rate (*γ*) causes a transition to stable coexistence, and further increase in herbivory is required to induce limit cycles again (Fig. 2). This makes sense because by speeding up the transition from larva to adult, the total effect of herbivory on the plants is reduced, hence preventing a crash in plant biomass followed by a crash in the insects. Third, when adult pollinators have alternative food sources (*ϕ* > 1), the zone of limit cycles in the space of parameters becomes larger (Fig. 4). This also makes sense, because the total effect of herbivory increases by an additional supply of larva (which is not limited by the nectar of the plant considered), leading to a plant biomass crash followed by insect decline.

The graphical analysis provides another indication that oscillations are herbivory driven. On the one hand insect isoclines (or rather larva isoclines) are always positively sloped, and insects only grow when plant biomass is large enough (how large depends on insect’s population size, due to intra-specific competition). Plant isoclines, on the other hand, can display a hump (Fig. 5B,C,D), and they grow (decrease) below (above) the hump. These two features of insect and plant isoclines are associated with limit cycles in classical victim–exploiter models [27]. If there is no herbivory or another form of antagonism (e.g. competition) but only mutualism, the plant’s isocline would be a positively sloped line, and plants would attain large populations in the presence of large insect populations, without cycles. However, mutualism is still essential for limit cycles: if mutualistic benefits are not large enough (*γσα* < *ην*), plant isoclines do not have a hump (Fig. 5A) and oscillations are predicted to vanish. The effect of mutualism on stability is like the effect of enrichment on the stability in pure victim–exploiter models [28], by allowing the plants to overcome the limits imposed by their intrinsic carrying capacity.

There is a minor *caveat* regarding our graphical analysis: whereas a hump in the plant’s isocline is a requisite for oscillations to evolve into limit cycles, this does not mean that isocline intersections at the left of the hump always lead to limit cycles (Fig. 5C). To our best knowledge, this always happens only for quite specific conditions in pure victim–exploiter models [40]. As long as we cannot prove by analytical means that intersection geometry determines local stability, the prediction of limit cycles remains a “rule of thumb”, based on extrapolating our knowledge about other victim–exploiter models.

### Classification of outcomes: mutualism or herbivory?

Interactions can be classified according to the net effect of one species on the abundance (biomass, density) of another (but see other schemes [41]). This classification scheme can be problematic in empirical contexts because reference baselines such as carrying capacities are usually not known [42].

Our PLA model illustrates the classification issue when non-equilibrium dynamics are generated endogenously, i.e. not by external perturbations. Since plants are facultative mutualists and insects are obligatory ones, one can say the outcome is *net mutualism* (+,+) or *net herbivory* (−,+), if the coexistence is stable, and the plant equilibrium ends up respectively above or below the carrying capacity [33, 34]. If coexistence is under non-equilibrium conditions and plant oscillations are entirely below the carrying capacity (e.g. for large herbivory rates), the outcome is detrimental for plants and hence there is net herbivory (−,+); oscillations may in fact be considered irrelevant for this conclusion (or may further support the case of herbivory, read below). However, when the plant oscillation maximum is above carrying capacity and the minimum is below, like in Fig. 3, could we say that the system alternates periodically between states of net mutualism and net herbivory? Here perhaps a time-based average over the cycle can help up us decide. The situation could be more complicated if plant oscillations lie entirely above the carrying capacity (see an example in S1 File): one can say that the net outcome is a mutualism due to enlarged plant biomasses, but the oscillations indicates that a victim–exploiter interaction exists. As we can see, deciding upon the net outcome require consideration of both equilibrium and dynamical aspects.

### Factors that could cause dynamical transitions

#### Environmental factors

The parameters in our analyses can change due to external factors. One of the most important is temperature [43]. It is well known, for example, that climate warming can reduce the number of days needed by larvae to complete their development [44], making larvae maturation rates (*γ*) higher. For insects that display Allee effects, a cooling of the environment will cause the sudden extinction of the insect and a catastrophic collapse of the mutualism, which cannot be simply reverted by warming. By retarding larva development into adults, cooling would increase the burden of herbivory over the benefits of pollination, making the system less stable by promoting oscillations. Flowering, pollination, herbivory, growth and mortality rates (e.g. *s*, *a*, *b*, *r*, *m* and *n* in equations 1) are also temperature-dependent and they can increase or decrease with warming depending on the thermal impacts on insect and plant metabolisms [45]. This makes general predictions more difficult. However, we get the general picture that warming or cooling can change the balance between costs and benefits impacting the stability of the plant–insect association.

Dynamical transitions can also be induced by changes in the chemical environment, often as a consequence of human activity. Some pesticides, for example, are hormone retarding agents [46]. This means that their release can reduce maturation rates, altering the balance of the interaction towards more herbivory and less pollination and finally endangering pollination service [47, 48]. In other cases, the chemical changes are initiated by the plants: in response to herbivory, many plants release predator attractants [49], which can increase larval mortality (*μ*). If the insect does nothing but harm, this is always an advantage. If the insect is also a very effective pollinator, the abuse of this strategy can cost the plant important pollination services because a dead herbivore today is one less pollinator tomorrow.

Another factor that can increase or decrease larvae maturation rates, is the level of nutrients present in the plant’s vegetative tissue [50, 51]. On the one hand, the use of fertilizers rich in phosphorus could increase larvae maturation rates [51]. On the other hand, under low protein consumption *M. sexta* larvae could decrease maturation rate, although *M. sexta* larvae can compensate this lack of proteins by increasing their herbivory levels (i.e. compensatory consumption) [50]. Thus, different external factors related to plant nutrients could indirectly trigger different larvae maturation rates that will potentially modify the interaction dynamics.

#### Pollinator’s diet breadth

An important factor that can affect the balance between mutualism and herbivory is the diet breadth of pollinators. Alternative food sources for the adults could lead to apparent competition [52] mediated by pollination, as predicted for the interaction between *D. wrigthii* (Solanacea) and *M. sexta* (Sphingidae) in the presence of *Agave palmieri* (plant) [6]: visitation of *Agave* by *M. sexta* does not affect the pollination benefits received by *D. wrightii*, but it increases oviposition rates on *D*. *wrightii*, increasing herbivory. As discussed before, such an increase in herbivory could explain why oscillations are more widespread when adult insects have alternative food sources (*ϕ* > 0) in our PLA model.

Although we did not explore this with our model, the diet breadth of the larva could also have important consequences. In the empirical systems that inspired our model, the larva can have alternative hosts [14], spreading the costs of herbivory over several species. The local extinction of such hosts could increase herbivory on the remaining ones, promoting unstable dynamics. To explore these issues properly, models like ours must be extended to consider larger community modules or networks, taking into account that there is a positive correlation between the diet breadths of larval and adult stages [7].

From the perspective of the plant, the lack of alternative pollinators could also lead to increased herbivory and loss of stability. The case of the tobacco plant (*N. attenuata*) and *M. sexta* is illustrative. These moths are nocturnal pollinators, and in response to herbivory by their larvae, the plants can change their phenology by opening flowers during the morning instead. Thus, oviposition and subsequent herbivory can be avoided, whereas pollination can still be performed by hummingbirds [11]. Although hummingbirds are thought to be less reliable pollinators than moths for several reasons [9], they are an alternative with negligible costs. Thus, a decline of hummingbird populations will render the herbivore avoidance strategy useless and plants would have no alternative but to be pollinated by insects with herbivorous larvae that promote oscillations.

### Conclusions

Many insect pollinators are herbivores during their larval phases. If pollination and herbivory targets the same plant (e.g. as between tobacco plants and hawkmoths), the overall outcome of the association depends on the balance between costs and benefits for the plant. As predicted by our plant-larva-adult (PLA) model, this balance is affected by changes in insect development: the faster larvae turns into adults the better for the plant and the interaction is more stable; the slower this development the poorer the outcome for the plant and the interaction is less stable (e.g. oscillations). Under plant–insect oscillations, this balance can be dynamically complex (e.g. periodic alternation between mutualism and antagonism). Since maturation rates play an essential role in long term stability, we predict important qualitative changes in the dynamics due to changes in environmental conditions, such as temperature and chemical compounds (e.g. toxins, hormones, plant nutrients). The stability of these mixed interactions can also be greatly affected by changes in the diet generalism of the pollinators.

## Supporting Information

S1 File Supplement containing the appendices cited in the main text. (PDF)

S1 Fig Detail of the *β* vs *γ* parameter space for specialist pollinators in the PLA model. The ellipse describes the joint variation of *γ* and *β* taking place in the bifurcation diagram in S2 Fig.
(EPS)

S2 Fig Bifurcation diagram for the PLA model. Parameters *γ* and *β* vary along the elliptical path drawn in S1 Fig, with reference for each quarter of a rotation. Solid (broken) lines represent stable (unstable) equilibria, black (white) circles represent limit cycle maxima and minima. The *x* = 1 line corresponds to the plant carrying capacity. *HB*_*super*_: super-critical and *HB*_*sub*_: sub-critical Hopf bifurcations, BP: branching point (transcritical bifurcation), LP: limit point (fold bifurcation).
(EPS)

S3 Fig Main configurations of the plant isocline. We only consider the O–K segment in the positive octant (hatched square). In A the isocline lies below the plant’s carrying capacity (i.e. left of K), in B parts of the isocline lie above (i.e. right of K).
(EPS)

S4 Fig Shape of the plant’s isocline. (A) As *γ* increases and *η*, *ν* decrease, points P and Q move closer to the diagonal asymptote (broken line), and the isocline eventually adopts the form of a mushroom. (B) As *β* increases, O, P, Q and the diagonal asymptote move towards the plant axis and the isocline is compressed vertically.
(EPS)

S5 Fig Main configurations of the larva isocline. The isocline consists of three black lines, but only the segment in the positive octant (hatched square) is biologically relevant. For A and B *ϕ* < *ν*. For C and D *ϕ* > *ν*. The green parabola *p*(*x*) is the numerator of the isocline and the circles indicate its roots, where *x*_0_: positive root. The red parabola *q*(*x*) is the denominator of the isocline, which has two roots *x* = 0 and *x* = *x*_*v*_, both of which are also the vertical asymptotes of the isocline. The isocline also has an horizontal asymptote *y*_*h*_. The alternative in part D can be dismissed because it implies a detrimental effect of plants on insects.
(EPS)

S6 Fig Shape of the larva isocline. (A) For *ϕ* < *ν* the larva isocline moves closer to the larva axis and becomes more shallow as *γ* and *β* increase. (B) For *ϕ* > *ν* the larva isocline becomes closer to the larva axis.
(EPS)

S7 Fig Plant oscillating above their carrying capacity in the PLA model. Blue:plant, green:larva, red:adult. The carrying capacity is indicated by the dotted line.
(EPS)

S8 Fig Oscillations in the PLA model started with different initial conditions (*). The oscillations can dampen out (blue) or converge to a limit cycle (red).
(EPS)

S9 Fig Interaction dynamics when flowers are explicitly considered. Blue:plant, green:larva, red:adult, black:flowers. The dotted line indicates the plant’s carryng capacity.
(EPS)
