# Supplementary material for: Dynamical Transitions in a Pollination–Herbivory Interaction: A Conflict between Mutualism and Antagonism
Source: PLoS One. 2015 Feb 20;10(2):e0117964. doi: 10.1371/journal.pone.0117964 (PMC4336290; doi:10.1371/journal.pone.0117964)
Supplement: S1 File — (PDF) [file pone.0117964.s001.pdf]

Supplement to “Dynamical transitions in a pollination–herbivory interaction: a conflict between mutualism and antagonism”

Tomás A. Revilla and Francisco Encinas-Viso

Appendix A: Bifurcations

Figure 1 in the main text shows all outcomes (plant-only, Allee effect, stable coexistence and limit cycles) occurring together in a rectangle at the bottom left corner of the parameter space β vs γ. We enlarged this rectangle in Figure S.1 in order to show the bifurcations of the PLA model as we traverse the parameter space along an elliptical path as indicated.

From Figure S.2 we can conclude that plant equilibrium biomasses (stable or not) are inversely related with the rate of herbivory (β). A similar response occurs regarding oscillations: as long as β values are large enough to induce oscillations (the part in the figure marked with circles), such oscillations tend to display lower maxima and minima for larger values of β, and higher maxima and minima for smaller values instead.

The response of plant biomasses with respect to the insect maturation rate (γ) is more complex. For example around the middle part of Figure S.2 (between the $\pi/2$ and $3\pi/2$ marks), increasing γ causes (equilibrium) plant biomass increases if herbivory is high, but decreases if herbivory is low. In contrast, increasing γ from very low values causes plant biomass to increase if herbivory is low (between LP and the $3\pi/2$ mark at the right) or decrease when it is high (between BP and the $\pi/2$ mark at the left).

The transitions between stability and limit cycles are typically *super-critical Hopf bifurcations*, in which a stable branch of periodic solutions overlaps a branch of unstable equilibria. The bifurcation diagram (Figure S.2) also displays a *sub-critical Hopf bifurcation*, in which an unstable branch of periodic solutions overlaps stable equilibria. In such cases the long term outcome can be stable coexistence or a limit cycle depending on the initial conditions. Given the parameter values in Table 1 of the main text, this sub-critical Hopf bifurcation zone was too narrow to be represented in the parameter space (Figure S.1). Appendix C contains a simulation in which a small change in the initial conditions causes the system to approach an equilibrium or a limit cycle.

The $R_o = 1$ line in Figure S.11 can be found analytically. To do this, we need to know when the carrying capacity equilibrium switches between stable and unstable, which depends on the eigenvalues of the jacobian matrix of the PLA model evaluated at $(x, y, z) = (1, 0, 0)$. The PLA model is:

$$\begin{aligned}
\frac{dx}{d\tau} &= x(1-x) + \sigma \frac{\alpha z}{\eta + z} x - \beta x y \\
\frac{dy}{d\tau} &= \epsilon \frac{\alpha x}{\eta + z} z + \phi z - \gamma \beta x y - \mu y \\
\frac{dz}{d\tau} &= \gamma \beta x y - \nu z
\end{aligned} \tag{A.1}$$

and its jacobian matrix at $(x, y, z) = (1, 0, 0)$ is:

$$\begin{bmatrix} 1 - 2x + \frac{\sigma\alpha z}{\eta+z} - \beta y & -\beta x & \frac{\sigma\alpha\eta x}{(\eta+z)^2} \\ \frac{\epsilon\alpha z}{\eta+z} - \gamma\beta y & -\mu - \gamma\beta x & \frac{\epsilon\alpha\eta x}{(\eta+z)^2} + \phi \\ \gamma\beta y & \gamma\beta x & -\nu \end{bmatrix} = \begin{bmatrix} -1 & -\beta & \frac{\sigma\alpha}{\eta} \\ 0 & -\mu - \gamma\beta & \frac{\epsilon\alpha}{\eta} + \phi \\ 0 & \gamma\beta & -\nu \end{bmatrix} \tag{A.2}$$

The eigenvalues of the jacobian are $\lambda_1 = -1$ and:

$$\lambda_2 = \frac{-(\mu + \nu + \gamma\beta) \pm \sqrt{(\mu + \nu + \gamma\beta)^2 - 4\left[\nu(\mu + \gamma\beta) - \gamma\beta(\phi + \epsilon\alpha/\eta)\right]}}{2}$$

thus $(x, y, z) = (1, 0, 0)$ is unstable if at least one of λ_2 have a positive real part. This can only happen when:

$$\frac{(\epsilon\alpha + \phi\eta)\gamma\beta}{\eta\nu(\mu + \gamma\beta)} > 1 \tag{A.3}$$

by which automatically both λ_2 are real (one is negative and the other is positive). The left-hand side of (A.3) is R_o in the main text. Making $R_o = 1$ and writing β as a function of γ, we obtain a decreasing hyperbolic line with asymptotes $\beta = 0$ and $\gamma = 0$ as shown in Figures 1 and 3 in the main text. This is yet another reason, a pure technical one this time, that explains why we choose to present our results in the form of a β vs γ parameter space.

Since the eigenvector of λ_1 is a multiple of $(1, 0, 0)$, the eigenvectors of λ_2 are orthogonal to $(1, 0, 0)$, i.e. $v = (0, v_y, v_z)$, $w = (0, w_y, w_z)$. This, and the fact that both λ_2 are real if the inequality above holds, means that only perturbations in y and/or z, i.e. an insect invasion, would make $(x, y, z) = (1, 0, 0)$ unstable.

Appendix B: Isocline properties

Let us assume that the adult phase is very short lived compared with the larval phase and with the dynamics of the plant. In the same way as we did in the case of the flowers, assume that the adults reach a steady-state $dz/dt \approx 0$ with respect to the other variables, and that the adult biomass can be approximated by $z \approx \gamma\beta xy/\nu$. Substituting this in the ODE system (A.1), we obtain the two-dimensional system:

$$\begin{aligned}\dot{x} &= x(1-x) + \sigma\frac{\alpha\gamma\beta x^2 y}{\eta\nu + \gamma\beta xy} - \beta xy \\ \dot{y} &= \epsilon\frac{\alpha\gamma\beta x^2 y}{\eta\nu + \gamma\beta xy} + \frac{\phi\gamma\beta xy}{\nu} - \gamma\beta xy - \mu y\end{aligned} \tag{B.1}$$

This system has two trivial isoclines, $x = 0$ for the plant and $y = 0$ for the insect. The following results only concern the non-trivial isoclines for plants and insects.

Plant isocline

Making $\dot{x} = 0$ in (B.1), the (non-trivial) isocline of the plant can be written as a polynomial in x and y:

$$x^2 y + \beta xy^2 - (1 + \sigma\alpha)xy + \frac{\eta\nu}{\gamma\beta}x + \frac{\eta\nu}{\gamma}y - \frac{\eta\nu}{\gamma\beta} = 0 \tag{B.2}$$

To characterize the shape of (B.2) we start by finding asymptotes. To do this we can rewrite (B.2) as a function of x:

$$y(x) = \frac{1}{2\beta}\left\{\frac{-\left(\frac{\eta\nu}{\gamma} - (1+\sigma\alpha)x + x^2\right) \pm \sqrt{\left(\frac{\eta\nu}{\gamma} - (1+\sigma\alpha)x + x^2\right)^2 + 4\frac{\eta\nu}{\gamma}x(1-x)}}{x}\right\} \tag{B.3}$$

We divide the numerator and the denominator of (B.3) by x:

$$\begin{aligned}y(x) &= \frac{1}{2\beta}\left\{-\frac{\eta\nu}{\gamma x} + (1+\sigma\alpha) - x \pm \sqrt{\frac{1}{x^2}\left(\frac{\eta\nu}{\gamma} - (1+\sigma\alpha)x + x^2\right)^2 + \frac{1}{x^2}4\frac{\eta\nu}{\gamma}x(1-x)}\right\} \\ &= \frac{1}{2\beta}\left\{-\frac{\eta\nu}{\gamma x} + (1+\sigma\alpha) - x \pm \sqrt{\left(\frac{\eta\nu}{\gamma x} - (1+\sigma\alpha) + x\right)^2 + 4\frac{\eta\nu}{\gamma}\left(\frac{1}{x} - 1\right)}\right\}\end{aligned}$$

and we take the limit when x goes to plus or minus infinity:

$$\begin{aligned}\lim_{x\to\pm\infty} y(x) &= \frac{1}{2\beta}\lim_{x\to\pm\infty}\left\{0 + (1+\sigma\alpha) - x \pm \sqrt{(0 - (1+\sigma\alpha) + x)^2 + 4\frac{\eta\nu}{\gamma}(0-1)}\right\} \\ &= \frac{1}{2\beta}\lim_{x\to\pm\infty}\left\{-(x - 1 - \sigma\alpha) \pm \sqrt{(x - 1 - \sigma\alpha)^2 - 4\frac{\eta\nu}{\gamma}}\right\}\end{aligned}$$

Note that $|x-1-\sigma\alpha| > \sqrt{(x-1-\sigma\alpha)^2 - 4\frac{\eta\nu}{\gamma}}$. Thus, the square root above can be approximated by $\delta(x)(x-1-\sigma\alpha)$, where δ is a number between 0 and 1, and $\delta(x) \to 1$ as $x \to \pm\infty$. We can continue as follows:

$$\begin{aligned}\lim_{x\to\pm\infty} y(x) &= \frac{1}{2\beta}\lim_{x\to\pm\infty}\{-(x-1-\sigma\alpha) \pm \delta(x)(x-1-\sigma\alpha)\} \\ &= \frac{x-1-\sigma\alpha}{\beta}\lim_{x\to\pm\infty}\frac{\{-1\pm\delta(x)\}}{2}\end{aligned} \tag{B.4}$$

When $x \to \pm\infty$ and $\delta \to 1$, the '+' branch, $y(x)$ approaches the horizontal asymptote $y = 0$. For this '+' branch we also have that $-1 < \{-1+\delta(x)\} < 0$ in (B.4), which means that y is negative when $x \to +\infty$, and positive when $x \to -\infty$. In other words, the horizontal asymptote is approached from below when $x \to +\infty$ and from above when $x \to -\infty$.

When $x \to \pm\infty$ and $\delta \to 1$, the '–' branch, $y(x)$ approaches the slanted asymptote:

$$y = \frac{1+\sigma\alpha - x}{\beta} \tag{B.5}$$

which decreases with x. For this '–' branch we also have that $-1 < \{-1-\delta(x)\}/2 < -1/2$ in (B.4), which means that when $x \to +\infty$, $y < 0$ and $|y| < |(x-1-\sigma\alpha)/\beta|$. In other words, y lies between 0 and the slanted asymptote when $x \to +\infty$.

If we write (B.2) as a function of y rather than as a function of x, we will find a vertical asymptote $x = 0$, and the slanted asymptote (B.5) again. Because (B.2) is symmetric regarding the signs of its terms, the properties of the vertical asymptote must consistent with those of the horizontal: $y(x)$ goes towards $+\infty$ when $x = 0$ is approached from the left, and towards $-\infty$ when $x = 0$ is approached from the right. Also because of symmetry x must lie between 0 and the slanted asymptote when $y \to +\infty$.

The following statements tells us the location of special points of (B.2) as well regions in which (B.2) cannot be satisfied.

Lemma 1: the plant isocline contains the following (x, y) points:

$$\begin{aligned}\mathrm{K} &= (1, 0) \\ \mathrm{O} &= (0, \beta^{-1}) \\ \mathrm{P} &= (\sigma\alpha - \eta\nu\gamma^{-1}, \beta^{-1}) \\ \mathrm{Q} &= (1, (\sigma\alpha - \eta\nu\gamma^{-1})\beta^{-1})\end{aligned} \tag{B.6}$$

Proof: evaluate (B.2) at $x = 1$ to get a quadratic equation in y with roots $y = 0$ and $y = (\sigma\alpha - \eta\nu/\gamma)/\beta$, this gives points K and Q respectively. Evaluate (B.2) at $y = \beta^{-1}$ to get a quadratic equation in x with roots $x = 0$ and $x = \sigma\alpha - \eta\nu/\gamma$, this gives points O and P respectively. Points K (the plant's carrying capacity), and O are always biologically feasible (both have non-negative coordinates).

Corollary 1: Simple observation of (B.6) tells us that points P and Q are simultaneously biologically feasible if $\gamma\sigma\alpha > \eta\nu$. Conversely, both are unfeasible if $\gamma\sigma\alpha < \eta\nu$.

Lemma 2: Points P and Q lie below the slanted asymptote (B.5).

Proof: substitute $y = \beta^{-1}$ in (B.5) to obtain point $(\sigma\alpha, \beta^{-1})$, and substitute $x = 1$ in (B.5) to obtain point $(1, \sigma\alpha/\beta)$. Simple inspection shows that point $(\sigma\alpha, \beta^{-1})$ is always to the right of point P, and point $(1, \sigma\alpha/\beta)$ is always above point Q.

Lemma 3: the plant isocline crosses the x- and y-axis only at points K and O respectively, and nowhere else.

Proof: substituting $y = 0$ in (B.2) gives only one root $x = 1$ (i.e. point K). Substituting $x = 0$ in (B.2) gives only one root $y = \beta^{-1}$ (i.e. point O).

Lemma 4: the plant isocline is not satisfied in the $(-,-)$ quadrant.

Proof: let $a, b \geq 0$ and substitute $x = -a$ and $y = -b$ in (B.2). This leads to:

$$-\left[a^2b + \beta ab^2 + (1+\sigma\alpha)ab + \frac{\eta\nu}{\gamma\beta}a + \frac{\eta\nu}{\gamma}b + \frac{\eta\nu}{\gamma\beta}\right] = 0 \tag{B.7}$$

since all parameter values are positive, the statement above is false, thus (B.2) is not satisfied in the $(-,-)$ quadrant.

Using this information about the asymptotes ($x = 0, y = 0$ and eq. B.5), and Lemmas 1, 2, 3 and 4 we can conclude that the plant's isocline must have one of the two forms depicted in figure S.3. Corollary 1 explains the form taken in figure S.3A, when $\gamma\sigma\alpha < \eta\nu$, and the form in figure S.3B, when $\gamma\sigma\alpha > \eta\nu$. These are the two main

cases referenced in the main text, where only the positive quadrant is considered. For points between the O–K segment and the axes $\dot{x} > 0$, otherwise $\dot{x} < 0$.

Figure S.4 shows how the positive part of the plant isocline changes as we vary some of the bifurcation parameters. Increasing γ or decreasing η or ν, causes the isocline to be "compressed" against the asymptote (B.5) and it adopts the shape of a mushroom, the letter Ω or an anvil. Increasing β causes points P and Q to decrease along the vertically axis. It is more difficult to follow the effect of the rest of the parameters, for example increasing σ and α cause P and Q to move right and upwards respectively, but they also move the asymptote (B.5) right and upwards, so we cannot tell if this will cause the isocline to adopt a mushroom shape.

Larva isocline

Making $\dot{y} = 0$ in (B.1) the larva isocline is:

$$y(x) = \frac{p(x)}{q(x)} \tag{B.8}$$

where the numerator and denominator:

$$p(x) = \epsilon\alpha\gamma\beta x^2 - \eta\nu\gamma\beta(1-\phi/\nu)x - \eta\mu\nu \tag{B.9}$$

$$q(x) = \gamma\beta\left[\gamma\beta(1-\phi/\nu)x + \mu\right]x \tag{B.10}$$

are second order polynomials, i.e. parabolas. By assuming instead $\dot{y} > 0$ one obtains (B.8) but with a ">" sign, which means that insect biomass grows for points lying below the isocline and conversely decline for points above the isocline.

For function $p(x)$: $p(0) = -\eta\mu\nu < 0$ and $\lim_{x\to\pm\infty} p(x) = +\infty$. This means that $p(x)$ has one negative root and one positive root; and also that $p(x) < 0$ between the negative and positive roots, and $p(x) > 0$ otherwise. Since $p(x)$ is the denominator of (B.8), the larva isocline has the same roots as $p(x)$ in the x-axis. The positive root of (B.9) and (B.8) is:

$$x_0 = \frac{\eta\nu}{2\epsilon\alpha}\left(1 - \frac{\phi}{\nu}\right) + \sqrt{\left[\frac{\eta\nu}{2\epsilon\alpha}\left(1-\frac{\phi}{\nu}\right)\right]^2 + \frac{\eta\mu\nu}{\epsilon\alpha\gamma\beta}} \tag{B.11}$$

For function $q(x)$: it has one root at $x = 0$, a second one at:

$$x_v = -\frac{\mu}{\gamma\beta(1-\phi/\nu)} \tag{B.12}$$

and $\lim_{x\to\pm\infty} p(x) = -\infty$. This means that $q(x) > 0$ between 0 and x_v, and $q(x) < 0$ otherwise. Both roots make the denominator of (B.8) equal to zero, which means that the larva isocline has two vertical asymptotes, $x = 0$ and x_v.

And finally, the larva isocline has one horizontal asymptote:

$$y_h = \lim_{x\to\pm\infty} \frac{p(x)}{q(x)} = \frac{\epsilon\alpha}{\gamma\beta(1-\phi/\nu)} \tag{B.13}$$

Notice that the signs of x_v and y_h depend on ϕ/ν:

$$\begin{cases} \phi < \nu: & x_v < 0, y_h > 0 \\ \phi > \nu: & x_v > 0, y_h < 0 \end{cases} \tag{B.14}$$

This information about the parabolas $(p(x), q(x))$, and the signs of the asymptotes (x_v, y_h), is enough to sketch the possible shapes of the larva isocline: the isocline crosses the x-axis at the roots of $p(x)$; its jumps to infinity at the roots of $q(x)$; and is positive (negative) whenever $p(x)$ and $q(x)$ have the same (different) signs. According to (B.14) we have two main cases:

1. If $\phi < \nu$ the vertical asymptote x_v is negative and the horizontal asymptote y_h is positive. As we can see, there are two alternatives, depicted by Figure S.5A and B. Both are indistinguishable in the positive octant, which is the only part that matters: they both start at the x_0 in the plant axis and grow up to a plateau y_h.

2. If $\phi > \nu$ the vertical asymptote x_v is positive and the horizontal asymptote y_h is negative. In this configuration we also have two alternatives, as depicted in Figures S.5C or D. However, we can quickly dismiss alternative D: the insect is meant to grow for points that are below the larva isocline, but since the isocline is decreasing, this automatically means to grow when plant abundance is low rather than high. This is nonsensical because the plant always has a positive effect on insects.

Figure S.6 shows how the positive part of the larva isocline responds to some parameter changes. From the equations that define the isocline's root (B.11) and asymptotes (B.12,B.13) we can conclude that increasing γ, β tends to move the isocline closer to the larva axis.

Appendix C: Additional simulations

Figure S.7 displays limit cycles in the PLA model with plant biomasses entirely above the carrying capacity. The parameters are as in Table 1 of the main text, but with $\gamma = 0.00973, \beta = 0.01$. Figure S.8 shows an example where oscillations can damped out or evolve towards a limit cycle depending on the initial conditions. Parameters as in Table 1 of the main text, but with $\gamma = 0.06, \beta = 20, \nu = 5$. The attraction basins for both outcomes are separated by an unstable orbit, like the one show in the bifurcation plot in Appendix A.

Figure S.9 displays the dynamics of plants, flowers, larva and adult insects under the interaction mechanism from which the PLA model is derived (ODE system 1 in the main text). This simulation uses parameter values from the last column of Table 1 of the main text with $\gamma = 0.01, b = 0.005$. This figure is comparable to Figure 2 in the main text: the 200 time in units there, become $t = \tau/r = 200/0.05 = 4000$ time units here, and the plant's carrying capacity there ($x = 1$), becomes $c^{-1} = 0.01^{-1} = 100$ here.

Appendix D: Source codes

Bifurcations plots

We used XPPAUT (`http://www.math.pitt.edu/~bard/xpp/xpp.html`) to generate the parameter spaces and bifurcation diagrams. Figures 1 and 3 of the main text and Figure S1 in this supplement were generated using with the script `'antmut.ode'`:

```
# Filename: antmut.ode
# Author: Tomas Revilla
# Description: This XPPAUT script integrates the PLA model using
# the default parameter values provided below. For bifurcation
# analysis change 'gamma=0.01' to 'gamma=0.009'in order to start
# with a stable system (no oscillations).

# Default parameter values used in Figures 1 and S1.
# For Figure 3, change 'phi=0' to 'phi=1'
par sigma=5
par epsilon=0.5
par alpha=5
par beta=10
par gamma=0.01
par eta=0.1
par mu=1
par nu=2
par phi=0

# Initial values
init x=1
init y=0.1
init z=0

# Settings
@ dt=0.001 bound=10000 total=500
```

```
@ yp1=x yp2=y yp3=z
@ ylo=0 yhi=10 xhi=500 nout=2000 nplot=3

# The following settings must be manually supplied
# to the AUTO module of XPPAUT
# nmax=500 ntst=30 ds=0.01 dsmin=0.001 dsmax=0.01
# epsu=0.0000001 epss=0.0000001 epsl=0.0000001

# Equations
x'=x*(1 - x) + (sigma*alpha*x*z)/(eta + z) - beta*x*y
y'=(epsilon*alpha*x*z)/(eta + z) + phi*z - gamma*beta*x*y - mu*y
z'=gamma*beta*x*y - nu*z
```

Figure S2 in this supplement was generated using with the script 'antmut_ellipse.ode', which is very similar to 'antmut.ode', except that parameters γ and β are constrained along the ellipse shown in Figure S1 in this supplement:

```
# Filename: antmut_ellipse.ode
# Author: Tomas Revilla
# Description: This XPPAUT script integrates the PLA model like
# 'antmut.ode', but parameters gamma and beta are constrained
# along an ellipse with the center and dimensions given below

# Default parameter values as in 'antmut.ode'
par sigma=5
par epsilon=0.5
par alpha=5
par eta=0.1
par mu=1
par nu=2
par phi=0
par angle=0

# Initial values
init x=1
init y=0.01
init z=0

# Ellipse parameters
number gammac=0.012
number betac=10
number width=0.004
number height=4
number phase=pi/2

# Elliptical path in beta vs gamma space
gamma=gammac + width*cos(-angle+pi)*cos(phase) - height*sin(-angle+pi)*sin(phase)
beta=betac + width*cos(-angle+pi)*sin(phase) + height*sin(-angle+pi)*cos(phase)

# To know which gamma and beta correspond to a given angle
aux gg=gamma aux bb=beta

# Settings
@ dt=0.001 bound=10000 total=500
@ yp1=x yp2=y yp3=z
@ ylo=0 yhi=10 xhi=500 nout=2000 nplot=3

# The following settings must be manually supplied
```

```
# to the AUTO module of XPPAUT
# nmax=500 ntst=30 ds=0.01 dsmin=0.001 dsmax=0.01
# epsu=0.0000001 epss=0.0000001 epsl=0.0000001

# Equations
x'=x*(1 - x) + (sigma*alpha*x*z)/(eta + z) - beta*x*y
y'=(epsilon*alpha*x*z)/(eta + z) + phi*z - gamma*beta*x*y - mu*y
z'=gamma*beta*x*y - nu*z
```

Numerical integration

We used the Runge-Kutta(4,5) method of Matlab (`https://www.mathworks.com/products/matlab/`) or Octave (`https://www.gnu.org/software/octave/`) to integrate the differential equations. The different ODE systems (PFLA , PLA, and PL models) were coded as ode-files, separated from the main scripts containing the parameter values and graphical settings.
Figure 3 in the main text and S7 in this supplement were generated by the script `'dynamics_tseries.m'` using the parameters and initial conditions listed inside:

```
% Filename: dynamics_tseries.m
% Author: Tomas Revilla
% Description: This script generates a time series of the scaled PLA model
% by numerical integration of the ode-file 'odepla.m'.
%
% Parameters (latin letters substitute greek symbols)
% s: sigma, pollination to plant biomass yield
% e: epsilon, pollination to insect biomass yield
% a: alpha, flower production respect to the plant's growth rate
% b: beta, herbivory respect to the plant's growth rate
% g: gamma, maturation rate, ONLY THIS ONE MUST BE LESS THAN 1
% h: eta, flower decay to utilisation ratio
% m: mu, larval mortality respect to the plant's growth rate
% n: nu, adult mortality respect to the plant's growth rate
% phi: insect intrinsic reproduction rate

clf
clear
global s a b g e h m n phi

s = 5;
e = 0.5;
a = 5;
b = 10;
g = 0.01; % change to 0.00973 for Figure S7 in this supplement
h = 0.1;
m = 1;
n = 2;
phi = 0;

n0 = [1, 0.01, 0];  % Initial conditions
tspan = 200; % change to 2000 for Figure S7 in this supplement

% Plant-Larva-Adult system
options = odeset('RelTol',1e-6,'AbsTol',[1e-6 1e-6 1e-6]);
[t, npla] = ode45(@odepla, [0, tspan], n0, options);

plot(t,npla)
xlabel('time')
```

```
ylabel('plant,larva,adult')
axis([0 tspan 0 2]) % change to [0 tspan 0 1.4] for Figure S7 in this supplement
hold on
line([0,tspan],[1,1],'Color', 'k', 'LineStyle', ':')
```

Figure S8 in this supplement was generated by the script 'dynamics_ucycle_3d.m' using the parameters and initial conditions listed inside:

```
% Filename: dynamics_ucycle_3d.m
% Author: Tomas Revilla
% Description: This script generates a time series of the scaled PLA model
% by numerical integration of the ode-file 'odepla.m'.
%
% Parameters (latin letters substitute greek symbols)
% s: sigma, pollination to plant biomass yield
% e: epsilon, pollination to insect biomass yield
% a: alpha, flower production respect to the plant's growth rate
% b: beta, herbivory respect to the plant's growth rate
% g: gamma, maturation rate, ONLY THIS ONE MUST BE LESS THAN 1
% h: eta, flower decay to utilisation ratio
% m: mu, larval mortality respect to the plant's growth rate
% n: nu, adult mortality respect to the plant's growth rate
% phi: insect intrinsic reproduction rate

clear
global s a b g e h m n phi

s = 5;
e = 0.5;
a = 5;
b = 20;
g = 0.06;
h = 0.1;
m = 1;
n = 5;
phi = 0;

n1 = [3.4, 0.9, 0.2]; % Initial condition for damped oscillations
n2 = [3.6, 0.9, 0.2]; % Initial condition for high amplitude oscillation

tspan = 100;

% Plant-Larva-Adult system
options = odeset('RelTol',1e-6,'AbsTol',[1e-6 1e-6 1e-6]);
[t, npla1] = ode45(@odepla, [0, tspan], n1, options);
[t, npla2] = ode45(@odepla, [0, tspan], n2, options);

clf

% orbits
plot3(npla1(:,1), npla1(:,3), npla1(:,2), 'b')
hold on
plot3(npla2(:,1), npla2(:,3), npla2(:,2), 'r')

% initial conditions
plot3(npla1(1,1), npla1(1,3), npla1(1,2), 'b*')
plot3(npla2(1,1), npla2(1,3), npla2(1,2), 'r*')

```

```
xlabel('plant')
zlabel('larva')
ylabel('adult')
grid on
```

The ode-file called by 'dynamics_tseries.m' and 'dynamics_ucycle_3d.m' to perform the numerical integration is 'odepla.m':

```
function dx = odepla(t,x)
% Filename: odepl.m
% Author: Tomas Revilla
% Description: Scaled Plant(1), Larva(2), Adult(3) model

global s a b g e h m n phi

dx = zeros(3,1);

dx(1) = x(1)*(1 - x(1)) + (s*a*x(1)*x(3))/(h + x(3)) -b*x(1)*x(2);
dx(2) = (e*a*x(1)*x(3))/(h + x(3)) + phi*x(3) -g*b*x(1)*x(2) -m*x(2);
dx(3) = g*b*x(1)*x(2) - n*x(3);

end
```

Figure 5 in the main text was generated by the script 'phase_space_pl.m' using the parameters and initial conditions listed inside:

```
% Filename: phase_space_pl.m
% Author: Tomas Revilla
% Description: This script generates four examples of phase-plane dynamics of
% the PL (plant, larva) model, a simplified version of the scaled PLA model.
%
% The model trajectories are obtained by numerical integration of the ode-file
% 'odepl.m'. The isoclines are drawn using the 'ezplot' function.
%
% Parameters (latin letters substitute greek symbols)
% s: sigma, pollination to plant biomass yield
% e: epsilon, pollination to insect biomass yield
% a: alpha, flower production respect to the plant's growth rate
% b: beta, herbivory respect to the plant's growth rate
% g: gamma, maturation rate
% h: eta, flower decay to utilisation ratio
% m: mu, larval mortality respect to the plant's growth rate
% n: nu, adult mortality respect to the plant's growth rate
% phi: insect intrinsic reproduction rate

clear clf global s a b g e h m n
tspan = 500;
options = odeset('RelTol',1e-6,'AbsTol',[1e-6 1e-6]);

%%%%%%%%%%%%%%%%%%%%%%%%%%%%%%%%%%%%%%%%%%%%%%%%%%%%%%%%%%%%%%%%%%%%%%%%%%%%%%%
% A: Net antagonism and damped oscillations
s = 3;
e = 0.7;
a = 3;
b = 10;
g = 0.02;
h = 0.1;
m = 1;
n = 2;
```

```
subplot(2,2,1)

% plant carrying capacity
line([1,1],[0,10],'Color', 'k', 'LineStyle', ':')
hold on

% plant and larva isoclines
piso = @(x,y) y*x^2 + b*x*y^2 -(1+s*a)*x*y + h*n*x/(g*b) + h*n*y/g -h*n/(g*b);
liso = @(x) (e*a*g*b*x^2 -h*n*g*b*x -h*m*n)/(g*b*x*(g*b*x + m));
ezplot(piso,[0,20,0,3])
ezplot(liso,[0,20,0,3])
title(['A␣(\gamma\sigma\alpha/\eta\nu=' num2str(g*s*a/(h*n)) ')'])
xlabel('')
ylabel('larva␣(y)')

% trajectories
n0 = [0.05, 0.12];
[t, npl] = ode45(@odepl, [0, tspan], n0, options);
plot(npl(:,1),npl(:,2), 'k-')
plot(n0(1),n0(2), 'k*')
n0 = [1.3, 0.01];
[t, npl] = ode45(@odepl, [0, tspan], n0, options);
plot(npl(:,1),npl(:,2), 'k-') plot(n0(1),n0(2), 'k*')
axis([0 1.5 0 0.15])
set(gca,'Xtick',[0.5, 1, 1.5],'XTickLabel',{'0.5', 'K=1', '1.5'})
set(gca,'Ytick',[0, 0.05, 0.1, 0.15],'YTickLabel',{'0', '0.05', '0.1', '0.15'})

%%%%%%%%%%%%%%%%%%%%%%%%%%%%%%%%%%%%%%%%%%%%%%%%%%%%%%%%%%%%%%%%%%%%%%%%%%%
% B: Net mutualism and Allee effect
s = 2.1;
e = 0.21;
a = 2;
b = 10;
g = 0.05;
h = 0.1;
m = 1;
n = 1.5;
subplot(2,2,2)

% plant carrying capacity
line([1,1],[0,10],'Color', 'k', 'LineStyle', ':')
hold on

% plant and larva isoclines
piso = @(x,y) y*x^2 + b*x*y^2 -(1+s*a)*x*y + h*n*x/(g*b) + h*n*y/g -h*n/(g*b);
liso = @(x) (e*a*g*b*x^2 -h*n*g*b*x -h*m*n)/(g*b*x*(g*b*x + m));
ezplot(piso,[0,20,0,3])
ezplot(liso,[0,20,0,3])
title(['B␣(\gamma\sigma\alpha/\eta\nu=' num2str(g*s*a/(h*n)) ')'])
xlabel('')
ylabel('')

% trajectories
n0 = [0.55, 0.14];
[t, npl] = ode45(@odepl, [0, tspan], n0, options);
plot(npl(:,1),npl(:,2), 'k-') plot(n0(1),n0(2), 'k*')
```

```
n0 = [0.8, 0.14];
[t, npl] = ode45(@odepl, [0, tspan], n0, options);
plot(npl(:,1),npl(:,2), 'k-') plot(n0(1),n0(2), 'k*')
n0 = [1.4, 0.05];
[t, npl] = ode45(@odepl, [0, tspan], n0, options);
plot(npl(:,1),npl(:,2), 'k-') plot(n0(1),n0(2), 'k*')
n0 = [1.4, 0.005];
[t, npl] = ode45(@odepl, [0, tspan], n0, options);
plot(npl(:,1),npl(:,2), 'k-') plot(n0(1),n0(2), 'k*')
axis([0 1.5 0 0.15])
set(gca,'Xtick',[0.5, 1, 1.5],'XTickLabel',{'0.5', 'K=1', '1.5'})
set(gca,'Ytick',[0, 0.05, 0.1, 0.15],'YTickLabel',{'0', '0.05', '0.1', '0.15'})

%%%%%%%%%%%%%%%%%%%%%%%%%%%%%%%%%%%%%%%%%%%%%%%%%%%%%%%%%%%%%%%%%%%%%%%%%%%%%%%%
% C: Mushroom shaped plant isocline, Allee effect and overexploitation
s = 3.7;
e = 0.2;
a = 3;
b = 10;
g = 0.02;
h = 0.1;
m = 1;
n = 1.5;
subplot(2,2,3)

% plant carrying capacity
line([1,1],[0,10],'Color', 'k', 'LineStyle', ':')
hold on

% plant and larva isoclines
piso = @(x,y) y*x^2 + b*x*y^2 -(1+s*a)*x*y + h*n*x/(g*b) + h*n*y/g -h*n/(g*b);
liso = @(x) (e*a*g*b*x^2 -h*n*g*b*x -h*m*n)/(g*b*x*(g*b*x + m));
ezplot(piso,[0,20,0,3])
ezplot(liso,[0,20,0,3])
title(['C␣(\gamma\sigma\alpha/\eta\nu=' num2str(g*s*a/(h*n)) ')'])
xlabel('plant␣(x)')
ylabel('larva␣(y)')

% trajectories
n0 = [5.5, 0.7]; [t, npl] = ode45(@odepl, [0, tspan], n0, options);
plot(npl(:,1),npl(:,2), 'k-')
plot(n0(1),n0(2), 'k*')
n0 = [4, 0.01]; [t, npl] = ode45(@odepl, [0, tspan], n0, options);
plot(npl(:,1),npl(:,2), 'k-')
plot(n0(1),n0(2), 'k*')
n0 = [2, 0.01]; [t, npl] = ode45(@odepl, [0, tspan], n0, options);
plot(npl(:,1),npl(:,2), 'k-')
plot(n0(1),n0(2), 'k*')
n0 = [1.1, 0.5]; [t, npl] = ode45(@odepl, [0, tspan], n0, options);
plot(npl(:,1),npl(:,2), 'k-')
plot(n0(1),n0(2), 'k*')
axis([0 6 0 1])
set(gca,'Xtick',[1, 2, 4, 6],'XTickLabel',{'K=1', '2', '4', '6'})

%%%%%%%%%%%%%%%%%%%%%%%%%%%%%%%%%%%%%%%%%%%%%%%%%%%%%%%%%%%%%%%%%%%%%%%%%%%%%%%%
% D: Mushroom shaped plant isocline, limit cycles
```

```
s = 5;
e = 0.3;
a = 5;
b = 10;
g = 0.02;
h = 0.1;
m = 1;
n = 2;
subplot(2,2,4)

% plant carrying capacity
line([1,1],[0,10],'Color', 'k', 'LineStyle', ':')
hold on

% plant and larva isoclines
piso = @(x,y) y*x^2 + b*x*y^2 -(1+s*a)*x*y + h*n*x/(g*b) + h*n*y/g -h*n/(g*b);
liso = @(x) (e*a*g*b*x^2 -h*n*g*b*x -h*m*n)/(g*b*x*(g*b*x + m));
ezplot(piso,[0,20,0,3])
ezplot(liso,[0,20,0,3])
title(['D␣(\gamma\sigma\alpha/\eta\nu=' num2str(g*s*a/(h*n)) ')'])
xlabel('plant␣(x)')
ylabel('')

% trajectories
n0 = [8, 0.01];
[t, npl] = ode45(@odepl, [0, tspan], n0, options);
plot(npl(:,1),npl(:,2), 'k-') plot(n0(1),n0(2), 'k*')
n0 = [3, 0.7];
[t, npl] = ode45(@odepl, [0, tspan], n0, options);
plot(npl(:,1),npl(:,2), 'k-') plot(n0(1),n0(2), 'k*')
n0 = [1.9, 1.9];
[t, npl] = ode45(@odepl, [0, tspan], n0, options);
plot(npl(:,1),npl(:,2), 'k-') plot(n0(1),n0(2), 'k*')
n0 = [0.01, 0.01];
% very close to cycle, to make it tick
[t, npl] = ode45(@odepl, [0, tspan], n0, options);
plot(npl(:,1),npl(:,2), 'k-', 'LineWidth',2)
axis([-0.1 20 -0.01 3])
set(gca,'Xtick',[1, 5, 10, 15, 20],'XTickLabel',{'K=1', '5', '10', '15', '20'})
```

The ode-file called by `'phase_space_pl.m'` to perform the numerical integration is `'odepl.m'`:

```
function dx = odepl(t,x)
% Filename: odepl.m
% Author: Tomas Revilla
% Description: Plant(1), Larva(2) model

global s a b g e h m n

dx = zeros(2,1);

dx(1) = x(1)*(1 - x(1)) +(s*a*g*b*x(2)*x(1)^2)/(h*n + g*b*x(1)*x(2)) -b*x(1)*x(2);
dx(2) = (e*a*g*b*x(2)*x(1)^2)/(h*n + g*b*x(1)*x(2)) -g*b*x(1)*x(2) -m*x(2);

end
```

Figure S9 in this supplement were generated by the script `'dynamics_pfla.m'` using the parameters and initial conditions listed inside:

```
% Filename: dynamics_pfla.m
% Author: Tomas Revilla
% Description: This script generates a time series of the PFLA model
% the un-scaled version with flowers present in the system. It calls
% the ode-file 'odepfla.m' for numerical integration
%
% Parameters
% r: plant intrinsic growth rate
% c: plant selfregulation coeff
% sigma: pollination to plant biomass yield
% a: pollination rate
% b: herbivory rate
% s: flower production rate
% w: flower decay rate
% gamma: maturation rate, ONLY THIS ONE MUST BE LESS THAN 1
% epsilon: pollination to insect biomass yield
% m: larval mortality rate
% n: adult mortality rate
% phi: insect intrinsic reproduction rate

clf
clear
global r c sigma a b s w epsilon gamma m n phi

r = 0.05;
c = 0.01;
sigma = 5;
a = 0.05;
b = 0.005;
s = 0.25;
w = 10*a;
epsilon = 0.5;
gamma = 0.01;
m = 0.05;
n = 0.1;
phi = 0.00;

n0 = [1/c, s/(w*c), 1, 0];  % Initial condition for the full system

tspan = 4000;

% Plant-Larva-Adult system
options = odeset('RelTol',1e-6,'AbsTol',[1e-6 1e-6 1e-6 1e-6]);
[t, npfla] = ode45(@odepfla, [0, tspan], n0, options);

% Copy without the flowers
npla = npfla(:,[1,3,4]);

clf
plot(t,npla) % plot everything but flowers
xlabel('time')
ylabel('plant,flower,larva,adult')
axis([0 tspan 0 2/c])
hold on
line([0,tspan],[1/c,1/c],'Color', 'k', 'LineStyle', ':')
plot(t,npfla(:,2), 'k'); % add the flowers as a black line
```

The ode-file called by 'dynamics_pfla.m' to perform the numerical integration is 'odepfla.m':

```
function dx = odepfla(t,x)
% Filename: odepfla.m
% Author: Tomas Revilla
% Description: unscaled Plant(1), Flower(2) Larva(3), Adult(4) model

global r c sigma a b s w epsilon gamma m n phi

dx = zeros(4,1);
dx(1) = r*x(1)*(1 - c*x(1)) + sigma*a*x(2)*x(4) - b*x(1)*x(3);
dx(2) = s*x(1) - w*x(2) - a*x(2)*x(4);
dx(3) = (epsilon*a*x(2) + phi)*x(4) - gamma*b*x(1)*x(3) - m*x(3);
dx(4) = gamma*b*x(1)*x(3) -n*x(4);
end
```

Supplementary figures

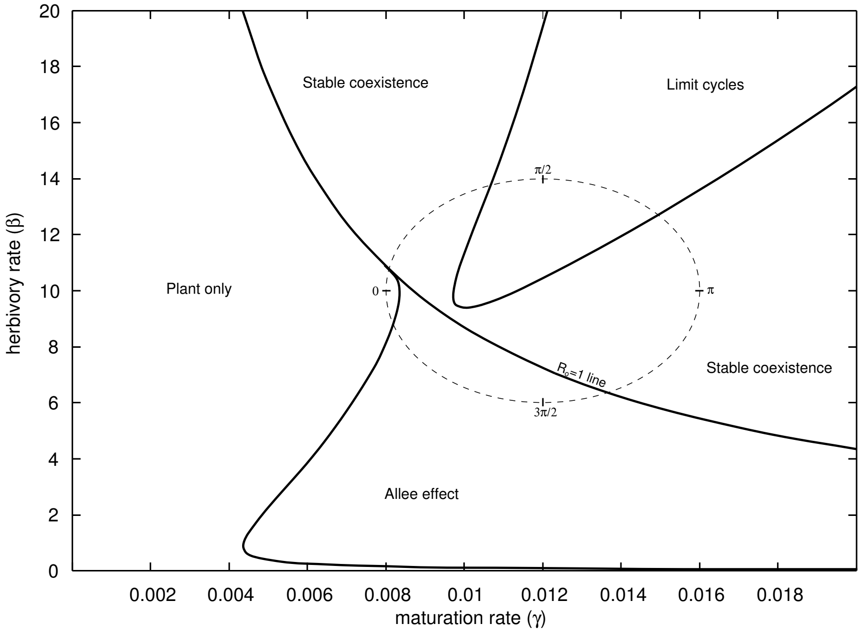

Figure S.1: Detail of the β vs γ parameter space for specialist pollinators in the PLA model. The ellipse describes the joint variation of γ and β taking place in the bifurcation diagram in Figure S.2.

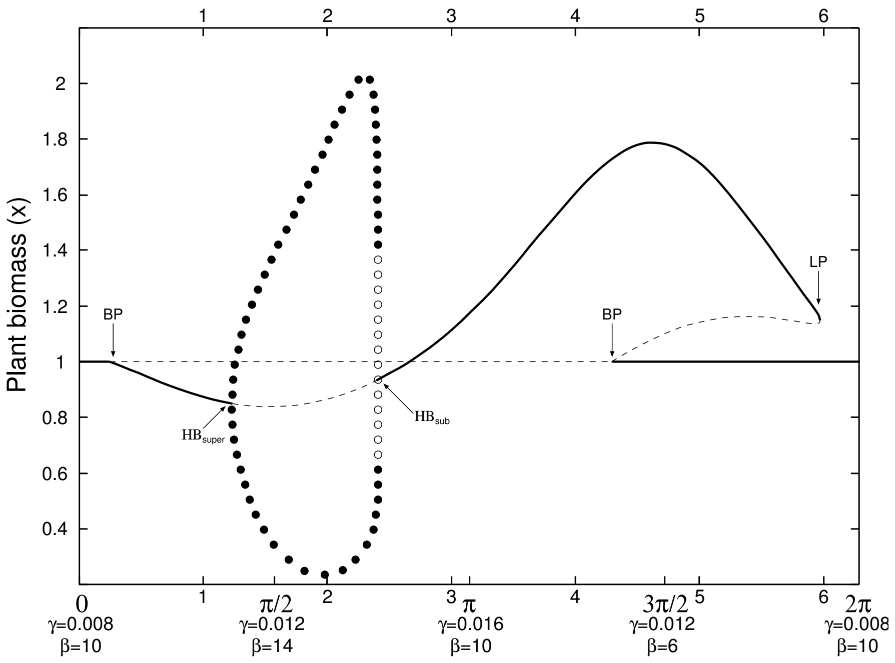

Figure S.2: Bifurcation diagram for the PLA model. Parameters γ and β vary along the elliptical path drawn in Figure S.1, with reference for each quarter of a rotation. Solid (broken) lines represent stable (unstable) equilibria, black (white) circles represent limit cycle maxima and minima. The $x = 1$ line corresponds to the plant carrying capacity. HB_{super}: super-critical and HB_{sub}: sub-critical Hopf bifurcations, BP: branching point (transcritical bifurcation), LP: limit point (fold bifurcation).

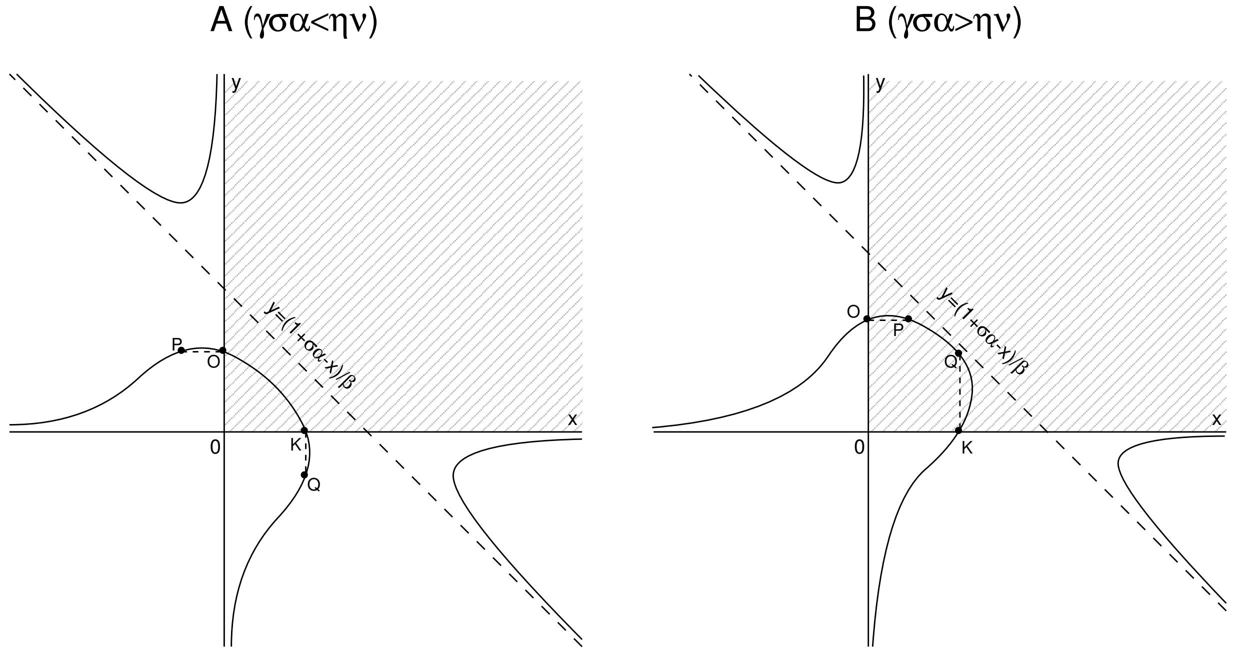

Figure S.3: Main configurations of the plant isocline. We only consider the O–K segment in the positive octant (hatched square). In A the isocline lies below the plant's carrying capacity (i.e. left of K), in B parts of the isocline lie above (i.e. right of K).

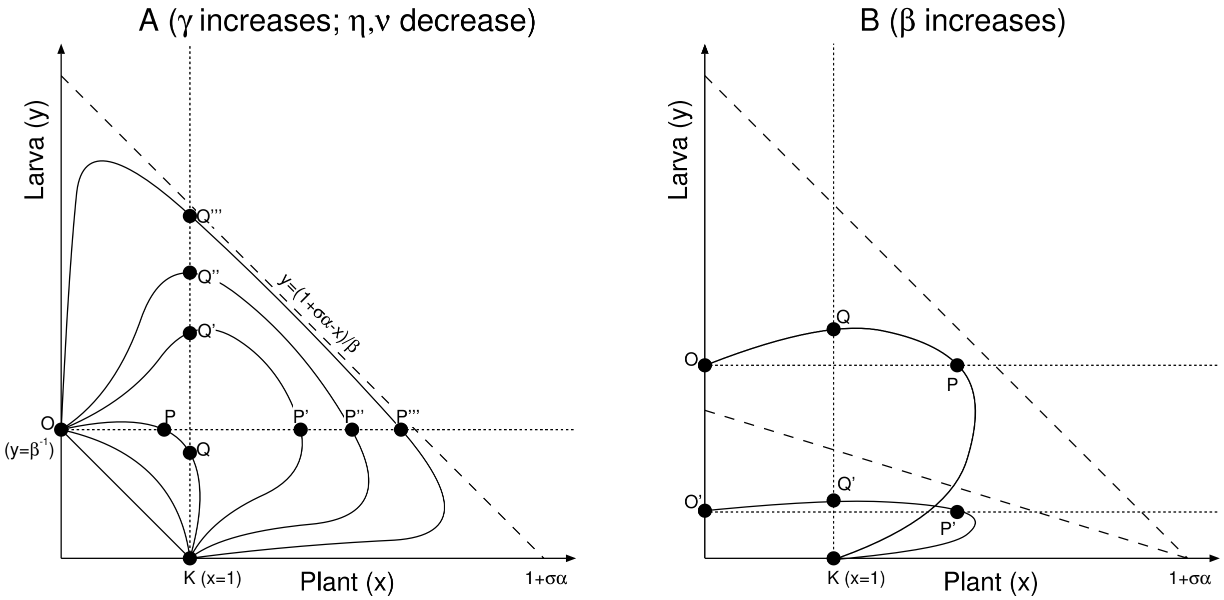

Figure S.4: Shape of the plant's isocline. (A) As γ increases and η, ν decrease, points P and Q move closer to the diagonal asymptote (broken line), and the isocline eventually adopts the form of a mushroom. (B) As β increases, O, P, Q and the diagonal asymptote move towards the plant axis and the isocline is compressed vertically.

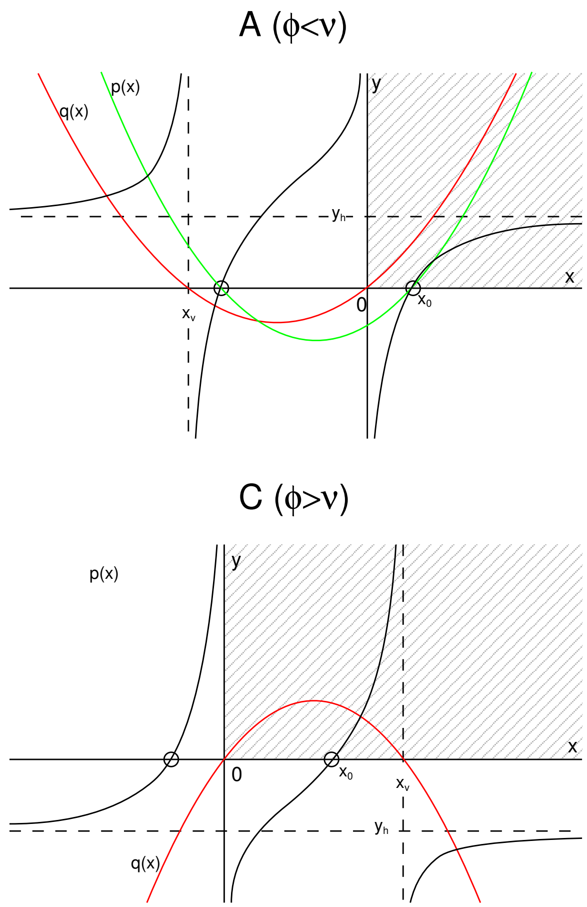

Figure S.5: Main configurations of the larva isocline. The isocline consists of three black lines, but only the segment in the positive octant (hatched square) is biologically relevant. For A and B $\phi < \nu$. For C and D $\phi > \nu$. The green parabola $p(x)$ is the numerator of the isocline and the circles indicate its roots, where x_0: positive root. The red parabola $q(x)$ is the denominator of the isocline, which has two roots $x = 0$ and $x = x_v$, both of which are also the vertical asymptotes of the isocline. The isocline also has an horizontal asymptote y_h. The alternative in part D can be dismissed because it implies a detrimental effect of plants on insects.

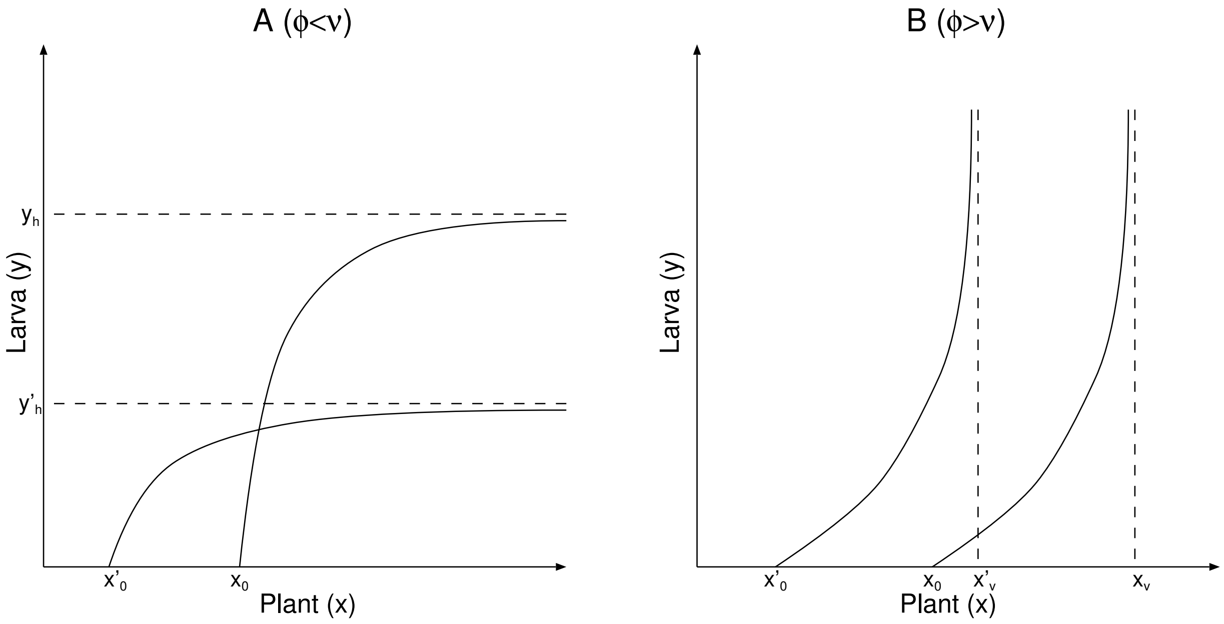

Figure S.6: Shape of the larva isocline. (A) For $\phi < \nu$ the larva isocline moves closer to the larva axis and becomes more shallow as γ and β increase. (B) For $\phi > \nu$ the larva isocline becomes closer to the larva axis.

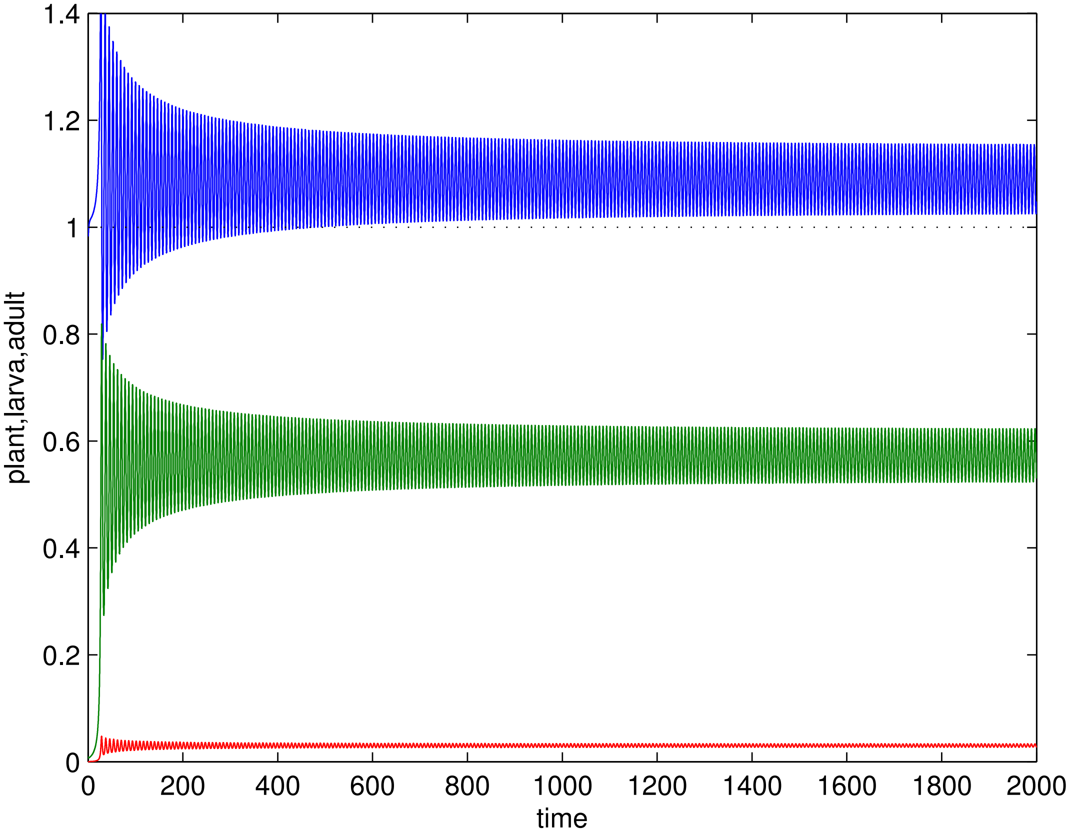

Figure S.7: Plants oscillating above their carrying capacity in the PLA model. Blue:plant, green:larva, red:adult. The carrying capacity is indicated by the dotted line.

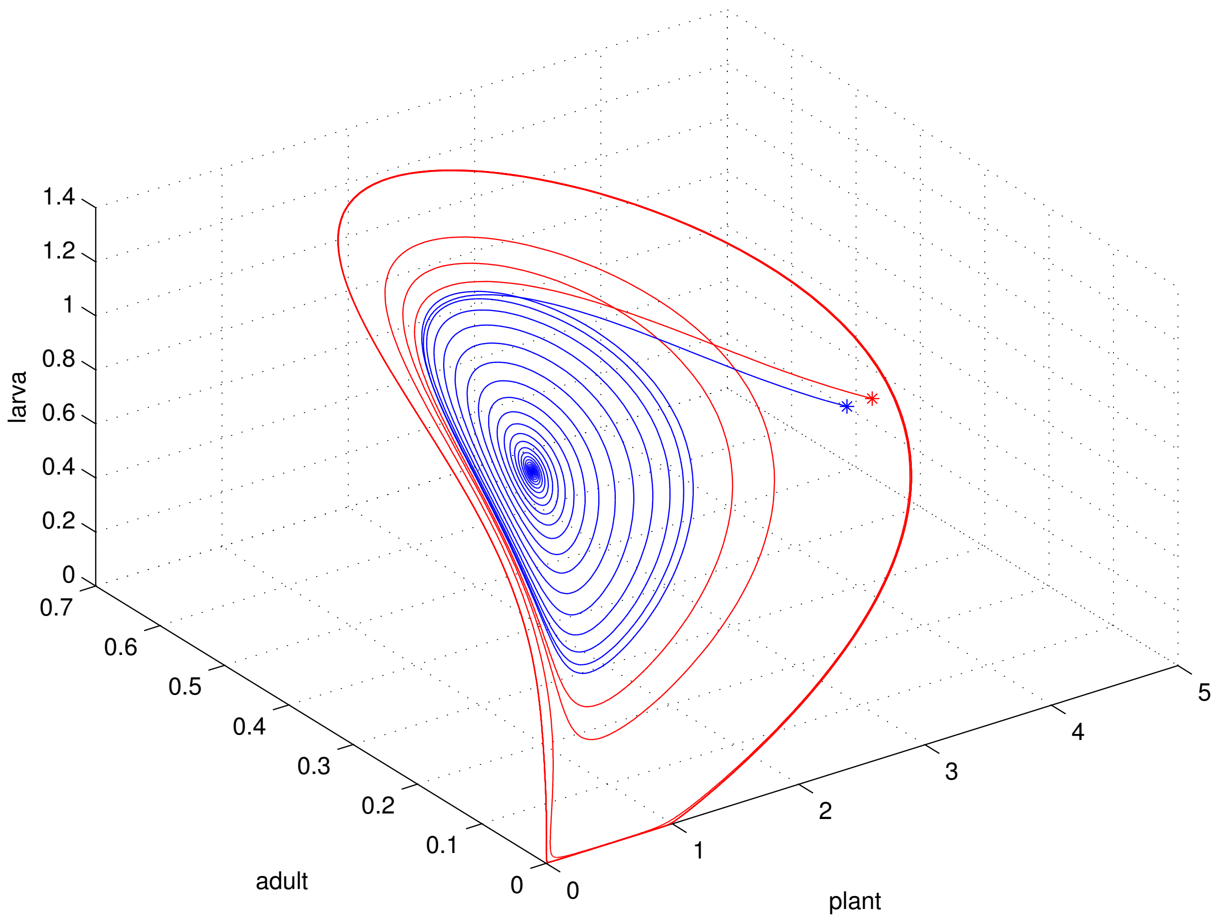

Figure S.8: Oscillations in the PLA model started with different initial conditions (*). The oscillations can dampen out (blue) or converge to a limit cycle (red).

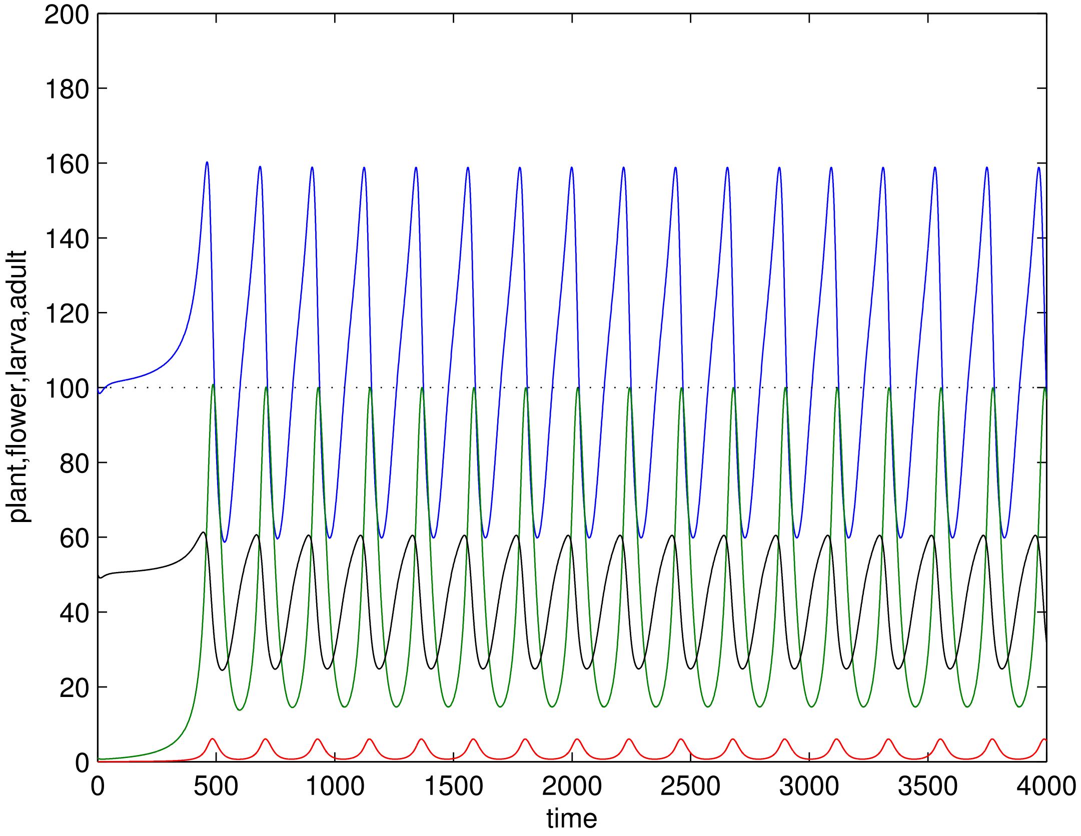

Figure S.9: Interaction dynamics when flowers are explicitly considered. Blue:plant, green:larva, red:adult, black:flowers. The dotted line indicates the plant's carryng capacity.
